# Mortality inequalities by occupational class among men in Japan, South Korea and eight European countries: a national register-based study, 1990–2015

**DOI:** 10.1136/jech-2018-211715

**Published:** 2019-05-29

**Authors:** Hirokazu Tanaka, Wilma J Nusselder, Matthias Bopp, Henrik Brønnum-Hansen, Ramune Kalediene, Jung Su Lee, Mall Leinsalu, Pekka Martikainen, Gwenn Menvielle, Yasuki Kobayashi, Johan P Mackenbach

**Affiliations:** 1 Department of Public Health, Erasmus University Medical Center, Rotterdam, The Netherlands; 2 Department of Public Health, Graduate School of Medicine, The University of Tokyo, Tokyo, Japan; 3 Epidemiology, Biostatistics and Prevention Institute, University of Zürich, Zürich, Switzerland; 4 Department of Public Health, University of Copenhagen, Copenhagen, Denmark; 5 Department of Health Management, Lithuanian University of Health Sciences, Kaunas, Lithuania; 6 Stockholm Centre for Health and Social Change, Södetörn University, Huddinge, Sweden; 7 Department of Epidemiology and Biostatistics, National Institute for Health Development, Tallinn, Estonia; 8 Population Research Unit, Department of Social Reseach, University of Helsinki, Helsinki, Finland; 9 INSERM, Institut Pierre Louis d'Epidémiologie et de Santé Publique, Sorbonne Universités, Paris, France

**Keywords:** epidemiology, cause of death/trends, socioeconomic factors, middle aged, registries

## Abstract

**Background:**

We compared mortality inequalities by occupational class in Japan and South Korea with those in European countries, in order to determine whether patterns are similar.

**Methods:**

National register-based data from Japan, South Korea and eight European countries (Finland, Denmark, England/Wales, France, Switzerland, Italy (Turin), Estonia, Lithuania) covering the period between 1990 and 2015 were collected and harmonised. We calculated age-standardised all-cause and cause-specific mortality among men aged 35–64 by occupational class and measured the magnitude of inequality with rate differences, rate ratios and the average inter-group difference.

**Results:**

Clear gradients in mortality were found in all European countries throughout the study period: manual workers had 1.6–2.5 times higher mortality than upper non-manual workers. However, in the most recent time-period, upper non-manual workers had higher mortality than manual workers in Japan and South Korea. This pattern emerged as a result of a rise in mortality among the upper non-manual group in Japan during the late 1990s, and in South Korea during the late 2000s, due to rising mortality from cancer and external causes (including suicide), in addition to strong mortality declines among lower non-manual and manual workers.

**Conclusion:**

Patterns of mortality by occupational class are remarkably different between European countries and Japan and South Korea. The recently observed patterns in the latter two countries may be related to a larger impact on the higher occupational classes of the economic crisis of the late 1990s and the late 2000s, respectively, and show that a high socioeconomic position does not guarantee better health.

## Introduction

Health inequalities between socioeconomic groups remain an important challenge for health and social policy around the world.^1^ Health inequalities are usually observed as a gradient, that is, a gradual, stepwise increase of morbidity and mortality among people lower on the social ladder.^2^ This suggests that the causes of inequalities in health are not simply poverty, or other unfavourable circumstances at the extremes of the social ladder, but factors that operate for everyone in society, such as psychosocial stress or social comparisons.^3^

Due to favourable behaviour changes and advances in prevention and treatment over the last 50 years, Japan and South Korea, the Asian member countries of the Organisation for Economic Cooperation and Development (OECD), have very long life expectancy. Thus, both countries have been recognised as global life expectancy leaders, together with European countries such as Switzerland, France, Spain and Italy.^4 5^ Although population health in Japan and South Korea shares many features with Western European countries, health inequalities in Japan and South Korea have sometimes been reported to be unique,^6–9^ with the highest socioeconomic groups not always having the best health, whereas in European countries inequalities in mortality and morbidity by socioeconomic position usually form steep and persistent gradients.^10–12^ Trend studies from Japan and South Korea have also shown remarkable trends, with rising all-cause mortality among managers and professionals after the late 1990s in Japan,^13 14^ and rising suicide mortality among managers after the late 2000s in South Korea.^15^

While some studies thus suggest that Japan and South Korea have unique patterns and trends of health inequalities, due to a lack of direct comparisons to other high-income countries this has remained uncertain.^6 9^ This study therefore aimed to systematically compare the magnitude and pattern of mortality inequalities by occupational class in Japan and South Korea with those in European OECD countries over the past 25 years. Documenting similarities and differences between two world regions will not only help to complete the global picture of health inequalities, but may also help to raise new hypotheses about the root causes of this phenomenon.

## Methods

### Data sources

We analysed national register-based data from eight European countries (Finland, Denmark, England/Wales, France, Switzerland, Italy (Turin), Estonia, Lithuania), Japan and South Korea. Our research group has collected mortality data by education and occupational class for a wide range of European countries,^10–12^ and the present study includes all European countries for which detailed mortality data by occupational class are available. Although mortality data for Italy came from Turin, an urban population in Northern Italy, previous studies have shown that patterns observed in Turin are similar to those observed at the national level.^16 17^

For European countries, information on occupational class for both the population denominator and the deceased were reported in the census.^10–12^ For Japan and South Korea, information on occupational class for the population denominator came from the census and that for the deceased was reported by the family on the death certificates.^14 18^ We used mortality data observed over 25 years divided into six periods: 1990–1994, 1995–1999, 2000–2004, 2005–2009, 2010–2014 and 2015 (the latter available for Japan and South Korea only) or similar. Underlying causes of death were classified according to the International Statistical Classification of Diseases (ICD, various revisions) and grouped into four broad groups (cancers, cardiovascular diseases, all other diseases and external causes), and eight specific causes of death known to be highly prevalent in Japan and South Korea. An overview of the data is presented in online supplementary appendix 1.

10.1136/jech-2018-211715.supp1 Supplementary data

### Occupational class

We categorised occupational class into five categories: upper non-manual workers (eg, professionals, managers), lower non-manual workers (eg, clerical, service, sales workers), manual workers (eg, craft and related trades workers, semi-skilled and unskilled manual workers), farmers and self-employed. This classification followed the Erikson-Goldthorpe-Portocarero scheme which was developed for international comparisons.^19^ The classification of specific occupations by occupational class is presented in online supplementary appendix 2, which also presents the educational composition of each occupational class in all countries included in the study except France. Online supplementary appendix table 2-1 presents the occupational class classification in Japan and South Korea; cross-national comparisons between European countries and East Asian countries have shown that despite some differences the patterns of social stratification and social mobility are largely similar.^20 21^ Because reliable occupational class data were not available for women and older men, the analyses will be restricted to men aged 35–64 years.

### Analysis

Age-standardised mortality rates (ASMR) by occupational class were computed using the 2013 European standard population and data in 5 year age intervals. In all countries except Finland, England/Wales and Italy (Turin), the last occupation was unknown for economically inactive men. This may cause bias, because economically inactive men tend to have higher mortality than economically active men, and because men in lower occupational classes have a higher likelihood of being economically inactive. For these countries, we therefore applied a previously developed and validated correction procedure (online supplementary appendix 3).^22–24^ Online supplementary appendix table 3-2 shows the percentages of men for whom occupational class was unknown in our dataset.

To measure inequality in mortality we computed rate differences (RDs) and rate ratios (RRs) by occupational class using upper non-manual workers as reference group. RDs were directly calculated as differences between the ASMRs of occupational classes. RRs adjusted for age and 95% CIs were estimated with Poisson regression. We also computed average inter-group differences (AIDs) as a summary measure of mortality inequality taking into account all occupational classes and their relative sizes.^25^ The AID has also been referred to as the ‘index of dissimilarity’, ‘index of disparity’ and ‘dispersion measure of mortality’.^26–28^ The AID (absolute version) was computed as the population weighted average of mortality differences within all pairs of occupational classes. For groups *i* and *j* (here, occupational class), the formula for the AID (absolute version) is:

­

(1) $$\begin{array}{ll} {\text{AID}}_{t}\left(\text{absolute version}\right) & =\frac{1}{2}\sum_{i=1}^{N}\sum_{j=1}^{N}\\ & \;\;\;\;\;\;\;\left|{\text{ASMR}}_{t,i}-{\text{ASMR}}_{t,j}\right|p_{t,i}p_{t,j} \end{array}$$

where *p_t,i_* and *p_t,j_* are the population shares of occupational class *i* and *j* in the total population (*i*, *j*=1, 2, …, *N*) at time *t*. In our analysis, *N*=5 (five occupational classes: upper non-manual, lower non-manual, manual workers, self-employed and farmers (four classes in some countries)). The AID (relative version) was computed as the AID (absolute version) divided by the average mortality rate in the whole male population aged 35–64 years. The AID (relative version) multiplied by 100 equals to the Gini coefficient which is often used as a measure of economic inequality in a population.^29^ The formula for the AID (relative version) is:

(2) $${\text{AID}}_{t}\left(\text{relative version}\right)\left(\%\right)=\frac{{\text{AID}}_{t}\left(\text{absolute}\text{version}\right)}{{\text{ASMR}}_{t}\left(\text{whole}\text{population}\right)}\times 100$$

The AID can be interpreted as the number or proportion of deaths that would have to be redistributed between occupational classes to achieve perfect equality.^26^

## Results

### Mortality inequality by occupational class

We observed a total of 1 570 708 deaths occurring in 293 370 858 person-years in Japan, South Korea and eight European countries combined over the whole study period between 1990 and 2015.

**Figure 1:**
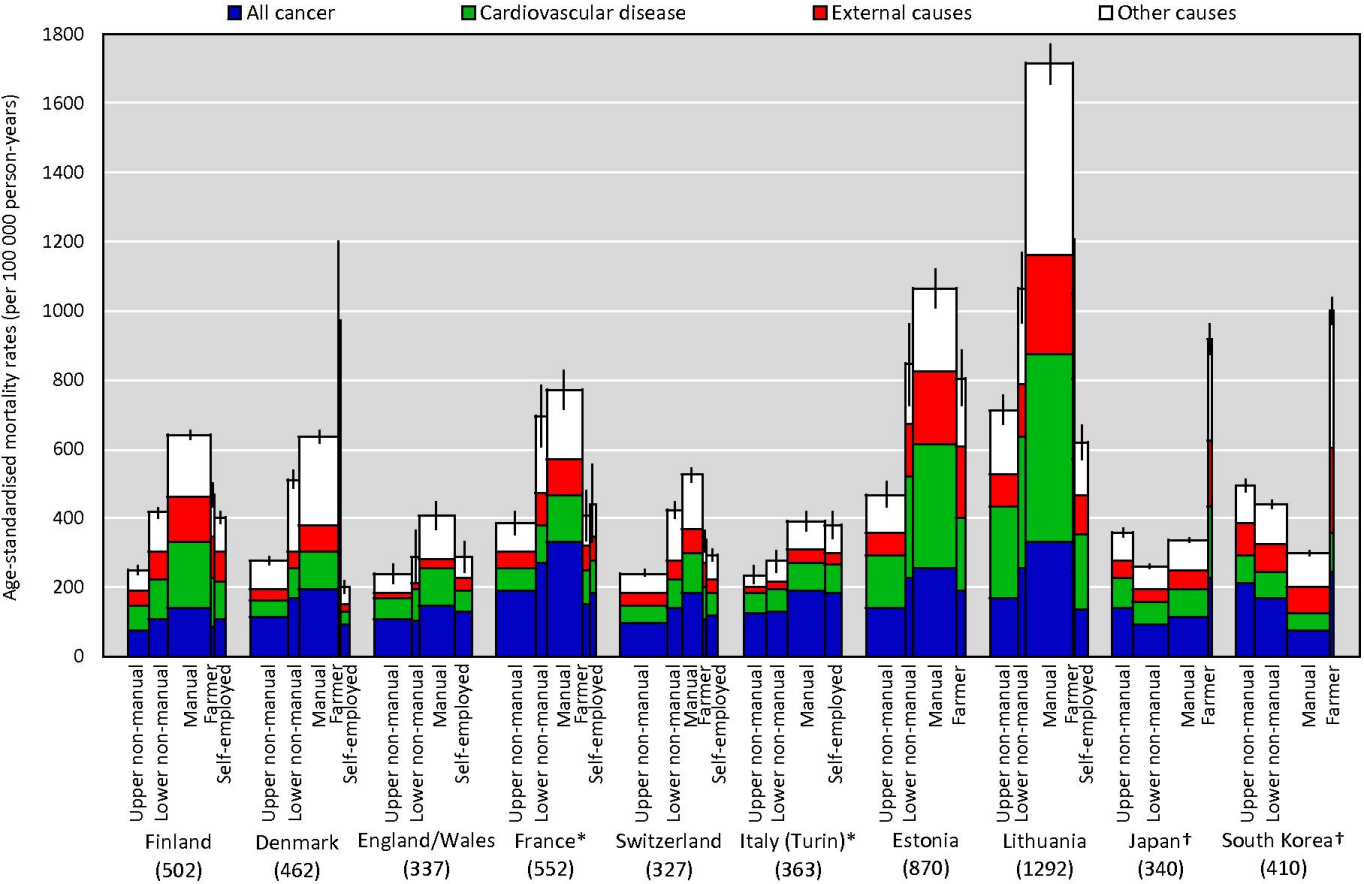
Age-standardised cause-specific mortality rate (95% CI) and population distribution by occupational class among men aged 35–64 in eight European countries, Japan and South Korea, 2010–2014 (2005–2009*, 2015†): number in parentheses indicate the whole population age-standardised all-cause mortality rate (per 100 000 person-years).

Figure 1 presents the ASMRs among men aged 35–64 by occupational class during the most recent period, with the height of bars indicating the mortality rate and the width of bars indicating the share of each occupational class in the population (online supplementary appendix table 4–1 to 4–10). The mortality rates in Japan and South Korea were comparatively low as they were in England/Wales, Switzerland and Italy (Turin), whereas they were very high in Estonia and Lithuania. Manual workers accounted for the largest percentages of population except in England/Wales, France and Switzerland where the upper non-manual workers were the largest group. The social gradient was clear and consistent in all European countries; that is, upper non-manual workers had the lowest mortality and manual workers had the highest mortality, with farmers and self-employed often having lower mortality than manual workers. However, the social gradient was different in Japan and South Korea; that is, farmers and upper non-manual workers had the highest and second highest mortality, respectively, whereas lower non-manual (Japan) and manual workers (South Korea) had the lowest mortality.

Table 1 shows absolute and relative inequalities in all-cause mortality as measured by RDs and RRs with upper non-manual workers as reference group in an earlier and in the most recent period. The RDs for manual workers have decreased in most European countries with the exception of Lithuania, whereas the RRs have sometimes decreased, and sometimes increased. However, in both Japan and South Korea the mortality disadvantage of manual workers has reversed into a mortality advantage: in Japan, the RR for manual workers changed from 1.13 (95% CI 1.10 to 1.15) in 1995 to 0.93 (95% CI 0.90 to 0.96) in 2015, and in South Korea the RR for manual workers changed from 2.48 (95% CI 2.39 to 2.57) in 1997 to 0.63 (95% CI 0.61 to 0.65) in 2015.

**Table 1:** Distribution of men (%), age-standardised all-cause mortality rate difference (RD)* and rate ratio (RR)† with their 95% CIs by occupational class‡

	1995–1999				2010–2014 (2005–2009§)					1995–1999 (2000–2004¶)				2010–2014 (2005–2009§, 2015**)			
	%	RD	RR	95% CI	%	RD	RR	95% CI		%	RD	RR	95% CI	%	RD	RR	95% CI
**Finland**									**Italy (Turin**)§								
Upper non-manual	17	0	1.00 (reference)		21	0	1.00 (reference)		Upper non-manual	18	0	1.00 (reference)		24	0	1.00 (reference)	
Lower non-manual	18	156	1.44	1.37 to 1.51	21	168	1.68	1.59 to 1.77	Lower non-manual	22	84	1.28	1.12 to 1.45	21	39	1.15	0.98 to 1.33
Manual	47	521	2.43	2.34 to 2.53	43	392	2.57	2.46 to 2.69	Manual	44	204	1.67	1.50 to 1.86	38	155	1.62	1.42 to 1.83
Farmers	8	242	1.68	1.59 to 1.77	4	221	1.83	1.70 to 1.98	Farmers	–	–	–	–	0.2	421	2.73	1.41 to 5.30
Self-employed	10	184	1.53	1.45 to 1.61	11	154	1.61	1.51 to 1.71	Self-employed	17	170	1.48	1.30 to 1.69	17	142	1.56	1.35 to 1.81
**Denmark**									**Estonia¶**								
Upper non-manual	35	0	1.00 (reference)		38	0	1.00 (reference)		Upper non-manual	34	0	1.00 (reference)		40	0	1.00 (reference)	
Lower non-manual	10	256	1.57	1.48 to 1.68	12	237	1.77	1.66 to 1.89	Lower non-manual	6	516	1.70	1.51 to 1.91	8	376	1.85	1.59 to 2.16
Manual	41	439	1.99	1.91 to 2.08	40	359	2.24	2.14 to 2.35	Manual	47	875	2.15	2.02 to 2.28	44	594	2.36	2.16 to 2.59
Farmers	1	303	1.81	1.56 to 2.11	1	698	3.25	2.53 to 4.18	Farmers	13	532	1.69	1.57 to 1.82	8	337	1.74	1.54 to 1.97
Self-employed	13	−65	0.86	0.81 to 0.91	9	−74	0.71	0.66 to 0.78	Self-employed	–	–	–	–	–	–	–	–
**England/Wales**									**Lithuania¶**								
Upper non-manual	7	0	1.00 (reference)		40	0	1.00 (reference)		Upper non-manual	22	0	1.00 (reference)		29	0	1.00 (reference)	
Lower non-manual	40	92	1.32	1.07 to 1.63	7	50	1.23	0.92 to 1.64	Lower non-manual	6	382	1.48	1.34 to 1.64	9	353	1.53	1.38 to 1.69
Manual	52	271	1.88	1.54 to 2.31	36	167	1.69	1.45 to 1.96	Manual	49	854	2.17	2.05 to 2.29	47	1001	2.43	2.28 to 2.59
Farmers	–	–	–	–	–	–	–	–	Farmers	5	960	2.29	2.12 to 2.47	2	336	1.54	1.32 to 1.79
Self-employed	–	–	–	–	18	49	1.20	0.99 to 1.46	Self-employed	18	624	1.82	1.71 to 1.94	13	−93	0.88	0.80 to 0.96
**France§**									Japan**								
Upper non-manual	38	0	1.00 (reference)		39	0	1.00 (reference)		Upper non-manual	20	0	1.00 (reference)		21	0	1.00 (reference)	
Lower non-manual	10	400	1.90	1.62 to 2.23	12	308	1.78	1.52 to 2.08	Lower non-manual	32	94	1.23	1.20 to 1.26	37	−99	0.71	0.69 to 0.73
Manual	36	544	2.24	2.00 to 2.50	35	385	1.99	1.78 to 2.23	Manual	43	43	1.13	1.10 to 1.15	40	−19	0.93	0.90 to 0.96
Farmers	10	137	1.29	1.09 to 1.53	9	24	1.01	0.84 to 1.22	Farmers	5	674	2.45	2.38 to 2.52	3	559	2.39	2.27 to 2.52
Self-employed	5	46	1.13	0.91 to 1.40	4	55	1.06	0.82 to 1.39	Self-employed	–	–	–	–	–	–	–	– –
**Switzerland**									South Korea**								
Upper non-manual	50	0	1.00 (reference)		48	0	1.00 (reference)		Upper non-manual	25	0	1.00 (reference)		22	0	1.00 (reference)	
Lower non-manual	16	242	1.63	1.57 to 1.70	15	182	1.72	1.62 to 1.83	Lower non-manual	23	1022	3.80	3.66 to 3.95	32	−57	0.92	0.89 to 0.95
Manual	17	408	2.05	1.98 to 2.13	20	284	2.17	2.06 to 2.28	Manual	36	436	2.48	2.39 to 2.57	44	−199	0.63	0.61 to 0.65
Farmers	5	59	1.17	1.10 to 1.24	4	95	1.40	1.27 to 1.53	Farmers	15	1011	3.97	3.82 to 4.12	4	502	1.96	1.88 to 2.06
Self-employed	13	105	1.27	1.22 to 1.32	12	53	1.22	1.15 to 1.30	Self-employed	–	–	–	–	–	–	–	–

*RDs were calculated using direct method with the 2013 European standard population.

†RRs and 95% CIs were estimated with Poisson regression adjusting age.

‡Results were applied to correction factors by countries and periods.

§France and Italy (Turin) in 2005–2009.

¶Estonia and Lithuania in 2000–2004.

**Japan and South Korea in 2015.

In the most recent period, cancer was the most important contributor (about 30%–35%) to inequalities in all-cause mortality between manual and upper non-manual workers in France, Switzerland and Italy (Turin), while cardiovascular disease was the most important contributor (about 30%–35%) in Finland, England/Wales, Estonia and Lithuania. In Japan and South Korea, cancer was by far the most important contributor (about 50%–80%) (data shown in online supplementary appendix table 4-11).

### Trends in mortality by occupational class

**Figure 2:**
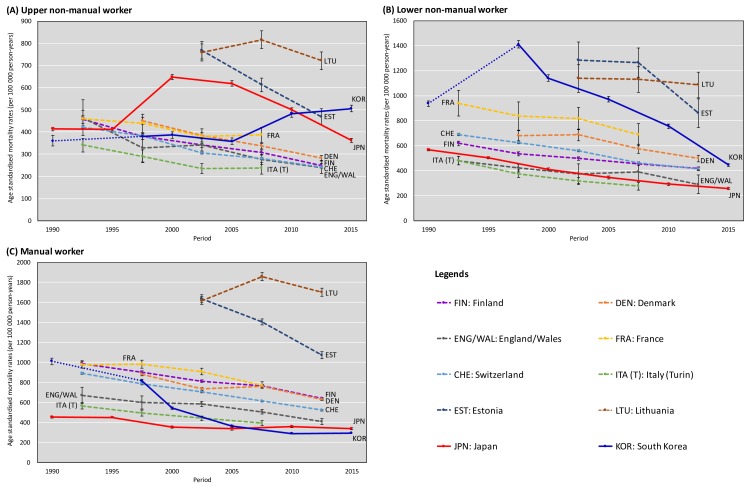
Trends in age-standardised all-cause mortality rates (95% CI) by occupational class (upper non-manual, lower manual and manual workers).

Figure 2 shows temporal trends in mortality by occupational class for upper non-manual, lower manual and manual workers. Mortality declined steadily in most European countries with the exception of Lithuania; however, in Japan and South Korea upper non-manual workers’ mortality rose after 2000 and after 2010, respectively. We observed consistently low and decreasing mortality among manual workers in Japan and very large mortality declines among both lower non-manual and manual workers in South Korea. The recently observed mortality patterns in Japan emerged during the late 1990s, and those in South Korea emerged during the late 2000s (see also online supplementary appendix figures 4-1 and 4-2).

Table 2 shows changes in mortality by cause of death among upper non-manual workers in Japan and South Korea. The increase in mortality in Japan in 1995–2000 was almost twice as large as the increase in mortality in South Korea in 2005–2010: all-cause mortality increased by 57% in Japan and 35% in South Korea. Many causes of death contributed to increasing mortality, but the largest percentage increases were seen for external causes (+118% in Japan (1995–2000) and +51% in South Korea (2005–2010)), particularly for suicide (+182% in Japan (1995–2000) and +93% in South Korea (2005–2010)). In absolute terms, cancer made the largest contribution to rising all-cause mortality both in Japan and South Korea in this occupational class.

**Table 2:** Changes in age-standardised all-cause and cause-specific mortality rate among upper non-manual workers in Japan and South Korea*

	1990–1995†		1995–2000†		2000–2005		2005–2010		2010–2015	
	ASMR‡ changes		ASMR‡ changes		ASMR‡ changes		ASMR‡ changes		ASMR‡ changes	
	Absolute	%§	Absolute	%§	Absolute	%§	Absolute	%§	Absolute	%§
**Japan**										
**All-cause**	−2	0	**236**	**57**	−28	−4	−119	−19	−139	−28
**Broad cause-specific death**										
All cancer	NA	–	**86**	**48**	−12	−4	−57	−22	−57	−29
Cardiovascular disease	NA	–	**45**	**47**	−5	−3	−26	−19	−25	−23
External causes	NA	–	**60**	**118**	−12	−11	−21	−21	−28	−35
Other causes	NA	–	**42**	**53**	3	2	−14	−11	−31	−28
**Cause-specific death**										
Stomach cancer	NA	–	**14**	**41**	−6	−12	−12	−26	−13	−39
Liver cancer	NA	–	6	16	−9	−20	−11	−31	−12	−46
Colorectal cancer	NA	–	**13**	**56**	0	−1	−4	−11	−9	−27
Ischaemic heart diseases	NA	–	**19**	**55**	−2	−4	−4	−9	−12	−25
Cerebrovascular diseases	NA	–	**21**	**48**	−9	−14	−12	−22	−11	−27
Smoking-related causes¶	NA	–	**19**	**62**	8	16	−11	−19	−15	−32
Suicide	NA	–	**54**	**182**	−8	−9	−14	−19	−21	−34
Road traffic accidents	NA	–	**6**	**56**	−4	−24	−5	−42	−2	−31
**South Korea**										
**All-cause**	20	5	6	2	−28	−7	**125**	**35**	22	5
**Broad cause-specific death**										
All cancer	NA	–	3	2	−10	−7	**44**	**30**	22	11
Cardiovascular disease	NA	–	−1	−2	−10	−13	10	16	5	7
External causes	NA	–	−1	−2	**9**	**16**	**33**	**51**	−2	−2
Other causes	NA	–	6	7	−18	−18	**37**	**46**	−8	−7
**Cause-specific death**										
Stomach cancer	NA	–	1	3	−7	−21	4	14	−4	−14
Liver cancer	NA	–	3	6	−5	−10	12	29	1	1
Colorectal cancer	NA	–	2	16	**5**	**42**	**7**	**43**	−1	−5
Ischaemic heart diseases	NA	–	**6**	**32**	−3	−10	7	29	0	0
Cerebrovascular diseases	NA	–	−3	−8	−7	−19	1	2	−4	−12
Smoking-related causes^¶^	NA	–	1	3	2	8	**15**	**49**	−1	−3
Suicide	NA	–	2	23	**20**	**171**	**29**	**93**	1	1
Road traffic accidents	NA	–	−3	−13	−8	−36	5	32	−2	−10

*The plus values (significant mortality increasing) were indicated by bold (p<0.05).

†1990–1997 and 1997–2000 in South Korea.

‡ASMR: age-standardised mortality rate (per 100 000 person-years).

§(ASMR changes)/(ASMR in previous study period)×100 (%)

¶Smoking-related causes: C33–34, J41–44 (ICD10) in Japan and C32–34, J40–44, J47 in South Korea.

ICD, International Statistical Classification of Diseases; NA, data not available.

### Mortality inequality assessed by the AIDs

**Figure 3:**
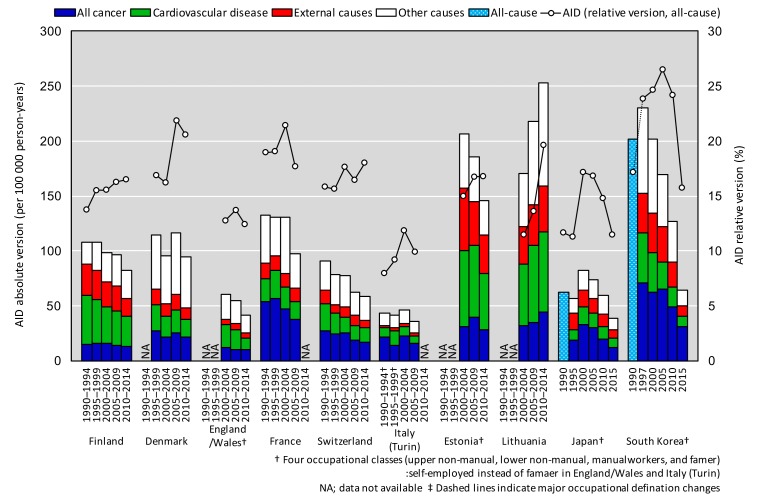
Changes in absolute and relative mortality inequalities (average inter-group difference: AID absolute and relative version), NA; data not available.

Figure 3 shows absolute and relative inequalities in mortality as measured by AIDs (see also online Supplementary Appendix Figure 4-3). In 2010–2014 the largest relative AIDs (between 16% and 24%) were found in Finland, Denmark, Switzerland, Estonia, Lithuania and South Korea, but inequalities in South Korea were declining steeply. Japan had comparatively small mortality inequalities both in absolute and relative terms except in 2000 and 2005. In all countries except Lithuania, absolute inequalities declined over time, whereas trends in relative inequalities showed a more variable pattern.

## Discussion

### Strengths and limitations

To the best of our knowledge, this is the first study which directly compared nation-wide inequalities in mortality between European countries, Japan and South Korea. Such a broad coverage, however, also increases the likelihood of comparability problems. Although we have carefully harmonised the data, some technical comparability problems remained. First, we were unable to distinguish the self-employed in Japan and South Korea (and in Estonia), because self-employment status was not registered on the death records. We therefore carried out a sensitivity analysis, using data on the proportion of self-employed by occupational class in Japan and South Korea from other sources, and the observed mortality rates among the self-employed as compared with the whole population in European countries. Although the self-employed are a heterogeneous category which cannot be assumed to represent exactly the same social group in all countries, the results (shown in online supplementary appendix 5) show that the pattern of mortality inequalities by occupational class in Japan and South Korea, including the higher mortality rate of upper non-manual group, is unlikely to be explained by lack of data on mortality of the self-employed.

Second, we had to apply a correction factor to take into account the varying proportions of men who were economically inactive during the census. Because mortality among the economically inactive was relatively high in Japan and South Korea, this had a large effect on the observed mortality patterns. However, the patterns observed in Japan and South Korea can already clearly be seen before the correction factor was applied (shown in online supplementary appendix figure 4–4).

Third, the data design in Japan and South Korea was cross-sectional unlinked, whereas that in European countries was longitudinal. A study comparing educational inequalities in mortality between a cross-sectional unlinked and a longitudinal design, showed that in the former design mortality rates among the high educated were underestimated, and mortality rates among the lower educated were overestimated.^30^ If similar differences would apply to mortality by occupational class, this would imply that in reality mortality among upper non-manual groups in Japan and South Korea are even higher, and that among manual groups even lower, than our results suggest.

Lastly, we focused on male mortality inequities in our analysis, because data on occupational class of women were often lacking. Future studies may exploit the strong increase of female workforce participation to investigate whether the patterns observed for men also apply to women.

### Interpretation

It is highly remarkable that the social gradient observed in all European countries, with manual workers consistently having the highest mortality and upper non-manual workers having the lowest mortality,^10–12 22–24^ is currently not found in Japan and South Korea. In addition, we also find very high mortality among farmers and extremely low mortality among manual workers in Japan and South Korea. Previous studies have identified some but not all of these patterns.^13 14 31^

These patterns of mortality in Japan and South Korea emerged only recently, due to an increase of mortality among upper non-manual workers and a continuing decline among manual workers. Although both countries experienced an increase, although it with a different timing, the increase (both absolute and relative term) observed in Japan was almost twice as large as that observed in South Korea. Other studies have shown that although South Korea also experienced the 1997 Asian financial economic crisis, the negative effect on mortality was limited.^32^ Meanwhile, despite the 2008 global financial crisis unfavourable mortality changes were not observed in European countries except in Lithuania.^33^

A possible mechanism underlying the rising mortality of upper non-manual workers is a change in their social work environment after the economic crises in Japan and South Korea. Japan’s economy underwent a long recession after the early 1990s, and the Japanese labour market changed as a result of ‘organisational streamlining and downsizing’ which caused an increase in working hours, particularly among men.^34^ Japanese managers have often been described as ‘playing managers’, because in this process they lost decision-making discretion while encountering ever greater psychological demands.^35^ It is suspected that similar unfavourable changes occurred in South Korea after the 2008 global financial crisis, as a result of increased psychological stress and job insecurity due to corporate downsizing and market restructuring. Whereas overtime hours of most other workers are decreasing due to social demands to reduce working hours, the work burden of managers has been increasing. Together with culture-specific responses to increased stress, such as feelings of shame, this may explain the increasing suicide mortality rates among upper non-manual workers in Japan and South Korea.^36 37^

However, it seems that other factors explain rising cancer mortality. Previous studies have suggested that Japanese managers and professional workers may not find the time for health check-ups due to their long working hours and heavy job demands, and therefore have high mortality from cancer.^13 14^ However, we could not find any statistics showing less health service utilisation among Japanese managers and professional workers. In any case, further study is needed to disclose why upper non-manual workers, who generally have more material (eg, income) and non-material (eg, social support or social participation) resources to cope with psychological and physical stress, were so vulnerable during the economic crisis in Japan and South Korea.

One possible explanation for the mortality patterns in Japan and South Korea is inconsistent and/or small differences between occupational groups in proximal risk factors for mortality. Several studies have shown upper class workers in Japan to have a relatively high prevalence of physical inactivity, high blood pressure and obesity.^38–40^ One study also found inequality in smoking prevalence among Japanese workers to be smaller than in other high-income countries.^41^ In South Korea, inequalities in unfavourable health behaviours and other proximal risk factors by occupational class were smaller than those by educational level.^42^ Another possible explanation is that the composition of occupational classes is different between Japan and South Korea and European countries. The group of manual workers consisted of over 20% of men with a high educational background (defined as International Standard Classification of Education 5 and 6) in Japan and South Korea, whereas the proportions were 10% or less in European countries (shown in online supplementary appendix table 2-2). This may have contributed to lower mortality among manual workers in Japan and South Korea, because level of education is a strong and consistent determinant of mortality in South Korea (unfortunately, there is little evidence on educational inequalities in mortality in Japan, due to a lack of educational information in Japanese vital statistics records).^18^

Previous studies have offered various other explanations for the patterns of health inequalities in Japan and South Korea. It has been suggested that Japan has smaller inequalities in mortality because of its smaller income inequalities and higher levels of social cohesion.^43^ However, according to OECD statistics, Japan does not really have smaller income inequalities than most European countries.^44^ Japan and South Korea also have different disease structures: as we have seen above, the contribution of specific diseases to mortality inequalities in Japan^14^ and South Korea^45^ is different from what we observe in other high-income countries.^10–12^ The larger share of cancer, for which socioeconomic inequalities in mortality tend to be smaller than for cardiovascular disease, may therefore contribute to smaller inequalities in all-cause mortality in Japan and South Korea.

There are also important differences in welfare policy and labour protection regulations between European countries and Japan and South Korea.^46 47^ The ‘welfare regime’ of Japan and South Korea has been characterised as ‘Confucian’, because of its greater emphasis on the role of the family and a stricter work ethic.^48 49^ For example, society is rather tolerant of overtime work, and moreover, managers often do unpaid overtime work in Japan and South Korea.^47 50^ It is possible that these differences have contributed to the differences in the health effects of economic crises on upper non-manual workers.

## Conclusions

Patterns of mortality by occupational class are remarkably different between European countries and Japan and South Korea. The recently observed patterns in the latter two countries call into question the often assumed universality of the relationship between socioeconomic position and health, and indicate that national context may act as an important modifier of this relationship. Further study of factors contributing to very low mortality among manual workers, and comparatively high mortality of upper non-manual workers, in Japan and South Korea, is necessary to shed more light on the explanation of these remarkable findings.

**What is already known on this topic**

- Inequalities in mortality and morbidity by socioeconomic position usually form steep and persistent gradients, but it is unclear whether this also applies to Japan and South Korea, two countries which have been recognised as global life expectancy leaders.
- Health inequalities in Japan and South Korea have sometimes been reported to be unique, with some studies reporting rising mortality among managers and professionals after the late 1990s in Japan and after the late 2000s in South Korea.
- However, this picture has remained uncertain due to a lack of direct comparisons to other high-income countries.

**What this study adds**

- In the most recent time period, male mortality was consistently higher in the manual group in eight European countries, but not in Japan and South Korea.
- The recently observed patterns in Japan and South Korea emerged during the last 25 years, as a result of rising mortality from cancer and external causes (particularly suicide) among the upper non-manual group, whereas mortality declined strongly among lower non-manual and manual groups.
- The timing of the mortality changes among the upper non-manual group in Japan and South Korea suggests that these were due to the economic crisis of the late 1990s (in Japan) and late 2000s (in South Korea).

**Policy implications**

- Japan has comparatively small mortality inequalities both in absolute and relative terms, whereas South Korea has been rapidly catching up with European countries and Japan due to strong mortality declines among lower non-manual and manual worker groups.
- The mortality inequality changes experienced in Japan and South Korea serve as a reminder that high socioeconomic position, which generally provides more material and non-material resources to cope with physical and psychological stress, does not guarantee better health, and that high mortality rates among skilled/unskilled manual worker groups are not inevitable.
