# Supplementary material for: Mortality inequalities by occupational class among men in Japan, South Korea and eight European countries: a national register-based study, 1990–2015
Source: J Epidemiol Community Health. 2019 May 29;73(8):750–8. doi: 10.1136/jech-2018-211715 (PMC6678055; doi:10.1136/jech-2018-211715)
Supplement: Supplementary data [file jech-2018-211715supp001.pdf]

Supplementary File

Tanaka, H. et al. Mortality inequalities by occupational class among men in Japan, South Korea, and 8 European countries: a national register-based study, 1990-2015

Contents:

Appendix 1 – Overview of data sources and mortality aged 35-64 men (page 2-5)

Appendix 2 – Definition of occupational class (page 6-10)

Appendix 3 – A procedure for adjusting estimates of occupational mortality differences for the exclusion of economically inactive men (page 11-13)

Appendix 4 – Age-standardized mortality rate (ASMR) by occupational class (page 14-31)

Appendix 5 – Sensitivity analysis using an indirect estimation of mortality among the self-employed in Japan and South Korea (page 32-34)

Appendix 1 – Overview of data sources and mortality aged 35-64 men

This appendix provides the descriptive statistics of data sources and mortality rate aged 35-64 men. Appendix Table 1-1 shows the overview of data sources and years covered by the analysis. Appendix Table 1-2 shows the distribution of person-years and deaths by occupational class, men aged 35-64. Cause-specific death was mainly defined as 4 types of broad cause-specific death according to International Statistical Classification of Diseases (ICD); all cancer (C00-D48), cardiovascular disease (I00-I99), external causes (V01-Y98), and other causes. Appendix Table 1-3 shows international statistical classification of diseases (ICD) codes "for the cause specific deaths.

Age-standardized all-cause mortality rate (ASMR) among men aged 35-64 by occupational class were computed per 100,000 person-years and directly standardized with the 2013 European standard population with data based on a 5-year age interval. Appendix Figure 1-1 shows trends in age-standardized all-cause mortality rate among the whole male population aged 35-64 years by countries.

Appendix Table 1-1. Overview of sources of mortality data

Population	Type of dataset	Observation period (Census year)						Geographic coverage	Demographic coverage
		1990-1994	1995-1999	2000-2004	2005-2009	2010-2014	2015		
**North Europe**									
Finland	Longitudinal, census linked	1990-1995<br>(1990)	1995-2000	2000-2005<br>(2000)	2005-2010	2010-2014[§]<br>(2010)	-<br>-	National	Whole population
Denmark	Longitudinal	-<br>-	1995-1999<br>(1995)	2000-2004<br>(2000)	2005-2009<br>(2005)	2010-2014<br>(2010)	-<br>-	National	Whole population
**West Europe**									
England/Wales	Longitudinal, census linked	1991-1996<br>(1991)	1996-2001	2001-2006<br>(2001)	2006-2011	2011-2013<br>(2011)	-<br>-	National	1% representative sample
France	Longitudinal, census linked	1990-1995<br>(1990)	1995-1999	1999-2004<br>(1999)	2004-2007	-<br>-	-<br>-	French-born in mainland	1% representative sample
Switzerland	Longitudinal, census linked	1990-1995<br>(1990)	1995-2000	2000-2005<br>(2000)	2005-2010	2010-2014<br>(2010)	-<br>-	National	Swiss nationals
**South Europe**									
Italy(Turin)	Longitudinal, census linked	1991-1996<br>(1991)	1996-2001	2001-2006<br>(2001)	2006-2010	-<br>-	-<br>-	City	Whole population
**Baltic country**									
Estonia	Longitudinal, census linked	-<br>-	-<br>-	2001-2006<br>(2001)	2006-2011	2012-2015<br>(2011)	-<br>-	National	Whole population
Lithuania	Longitudinal, census linked	-<br>-	-<br>-	2001-2005<br>(2001)	2005-2009	2011-2014<br>(2011)	-<br>-	National	Whole population
**Asia**									
Japan*	Cross-sectional, census unlinked	1990[†]<br>(1990)	1995<br>(1995)	2000<br>(2000)	2005<br>(2005)	2010<br>(2010)	2015<br>(2015)	National	Japanese nationals
South Korea*	Cross-sectional, census unlinked	1990[†]<br>(1990)	1997<br>(1995)	2000<br>(2000)	2005<br>(2005)	2010<br>(2010)	2015<br>(2015)	National	South Korea nationals

* The national census was conducted every 5 years on 1 October in Japan and on 1 November in South Korea, respectively.

† All-cause of death only

§ 80% representative sample

Appendix Table 1-2. Distribution of person-years and number of deaths by occupational class, men aged 35-64

		1990-1994		1995-1999		2000-2004		2005-2009		2010-2014		2015	
		Person-years	Deaths	Person-years	Deaths	Person-years	Deaths	Person-years	Deaths	Person-years	Deaths	Person-years	Deaths
Finland	Upper non-manual	818206	2925	819351	2907	963414	3132	915425	3110	881923	2177	-	-
	Lower non-manual	871407	4586	857155	4442	1037365	4884	984131	4824	854377	3582	-	-
	Manual	2337154	20882	2203329	19311	2469452	19423	2282735	19211	1791177	12899	*Data not available*	
	Farmers	434791	3333	359689	2565	311787	1917	260978	1666	159648	950	-	-
	Self-employed	477029	2759	450461	2563	540128	2748	487098	2904	470014	2139	-	-
	Inactive or class unknown	67116	875	73230	795	163575	1440	178173	1574	182989	1566	-	-
Denmark	Upper non-manual	-	-	1388237	3396	1446184	3015	1531016	2911	1698368	2735	-	-
	Lower non-manual	-	-	402844	1272	349068	1010	366098	934	541308	1428	-	-
	Manual	*Data not available*		1657350	5727	2093399	6610	1458143	4330	1777331	4524	*Data not available*	
	Farmers	-	-	59446	177	35737	88	22425	65	24183	62	-	-
	Self-employed	-	-	504860	1795	503163	1580	505948	1307	387802	755	-	-
	Inactive or class unknown	-	-	1171106	19937	1134687	19262	1843199	21110	1245851	16722	-	-
England/Wales	Upper non-manual	32973	130	32858	100	206070	639	198872	569	118741	272	-	-
	Lower non-manual	188594	810	183910	759	26561	92	25966	102	19770	57	-	-
	Manual	250157	1651	237521	1467	197045	1144	181146	952	106766	451	*Data not available*	
	Farmers	-	-	-	-	-	-	-	-	-	-	-	-
	Self-employed	-	-	-	-	80787	349	71786	296	52879	160	-	-
	Inactive or class unknown	15014	183	14780	167	18000	161	17651	143	15169	111	-	-
France	Upper non-manual	153762	566	122834	476	178225	546	140101	513	-	-	-	-
	Lower non-manual	43070	328	33092	222	53632	335	41593	235	-	-	-	-
	Manual	148714	1153	118189	941	158180	985	125904	753	*Data not available*		*Data not available*	
	Farmers	45346	205	33670	191	45632	176	33656	139	-	-	-	-
	Self-employed	27342	147	17791	101	20266	84	14442	62	-	-	-	-
	Inactive or class unknown	20546	480	13337	310	31461	560	21168	349	-	-	-	-
Switzerland	Upper non-manual	2250925	7447	2189646	7866	2146281	4485	2089066	5120	1687463	3790	-	-
	Lower non-manual	698427	3561	685911	3669	554922	1738	551209	1861	531156	1550	-	-
	Manual	802439	5398	748964	5117	754420	3053	726046	3281	709737	2695	*Data not available*	
	Farmers	258816	1111	232840	1077	214443	650	198254	660	157153	518	-	-
	Self-employed	598998	2897	556906	2833	629855	2098	573368	2116	420364	1496	-	-
	Inactive or class unknown	303089	5954	225461	3027	1249757	11547	1020064	8093	951142	5513	-	-
Italy(Turin)	Upper non-manual	151194	476	126670	418	179587	413	128123	336	-	-	-	-
	Lower non-manual	175722	767	153050	575	155726	483	111898	331	-	-	-	-
	Manual	394583	2504	308718	1842	296295	1368	203911	863	*Data not available*		*Data not available*	
	Farmers	-	-	-	-	2056	11	1321	9	-	-	-	-
	Self-employed	148845	771	119075	640	135321	493	90873	378	-	-	-	-
	Inactive or class unknown	40355	649	25147	297	63109	507	44101	349	-	-	-	-
Estonia	Upper non-manual	-	-	-	-	326235	1441	321468	1548	270562	678	-	-
	Lower non-manual	-	-	-	-	60421	329	62719	466	55215	217	-	-
	Manual	*Data not available*		*Data not available*		443518	3360	414477	3931	294486	1398	*Data not available*	
	Farmers	-	-	-	-	118743	1338	102733	1226	52954	383	-	-
	Self-employed	-	-	-	-	-	-	-	-	-	-	-	-
	Inactive or class unknown	-	-	-	-	414813	11538	313183	7150	299088	5633	-	-
Lithuania	Upper non-manual	-	-	-	-	399955	1571	339200	1697	406915	1296	-	-
	Lower non-manual	-	-	-	-	106520	478	101951	519	120413	496	-	-
	Manual	*Data not available*		*Data not available*		869923	6027	722667	6879	661278	3711	*Data not available*	
	Farmers	-	-	-	-	92640	1184	73838	1207	28288	195	-	-
	Self-employed	-	-	-	-	317142	2926	266083	3045	177685	716	-	-
	Inactive or class unknown	-	-	-	-	1166075	28167	828386	22202	812394	21284	-	-
Japan	Upper non-manual	4181083	11397	4561911	11521	4137684	14901	3783755	11414	3857965	9236	3978555	6510
	Lower non-manual	7014302	22158	7240800	20504	7547904	17761	7482713	13779	7293419	10968	7035741	8310
	Manual	9960732	23510	9677262	21815	9126617	15387	8865972	13009	8105406	11293	7708286	9473
	Farmers	1456445	9570	1089070	6891	801737	4593	717228	3445	650675	2935	549320	1945
	Self-employed	-	-	-	-	-	-	-	-	-	-	-	-
	Inactive or class unknown	2047748	57106	2276364	63237	2921203	67896	4313701	74867	5841310	74886	5532195	59102
South Korea	Upper non-manual	602552	1180	1702210	3618	1845991	3747	1890626	3597	1921042	4381	1966422	5470
	Lower non-manual	1938826	10349	1557672	11114	1851810	10092	2294428	9995	2686796	9503	3189323	7818
	Manual	1760799	8481	2439444	12387	2656940	8778	3037999	6668	3682534	6451	4301190	9173
	Farmers	1204764	19006	994586	14389	807825	11508	640889	6542	494120	3850	388094	3049
	Self-employed	-	-	-	-	-	-	-	-	-	-	-	-
	Inactive or class unknown	794792	21197	856899	17845	1428226	23734	1676234	24259	1670055	24734	1729868	19974

Appendix Table 1-3. International Statistical Classification of Diseases (ICD) codes for the cause-specific death

Cause of death	ICD-10	
	European countries and South Korea	Japan
**Broad cause-specific death**		
All Cancer	C00-D48	C00-C97
Cardiovascular disease	I00-I99	I01-I02.0, I10-I13, I05-I09, I20-I25, I27, I30-I51, I60-I69, I71
External causes	V01-Y98	V01-X84
Other causes	Others disease	Others disease
**Cause-specific death**		
Stomach cancer	C16	C16
Liver cancer	C22	C22
Colorectal cancer	C18-C21	C18-C20
Ischemic heart diseases	I20-I25	I20-I25
Cerebrovascular diseases	I60-I69	I60-I69
Smoking related causes	C32-34, J40-44, J47	C33-34, J41-44
Suicide	X60-X84, Y87.0	X60-X84
Road traffic accidents	V01-V89, Y85	V01-V98

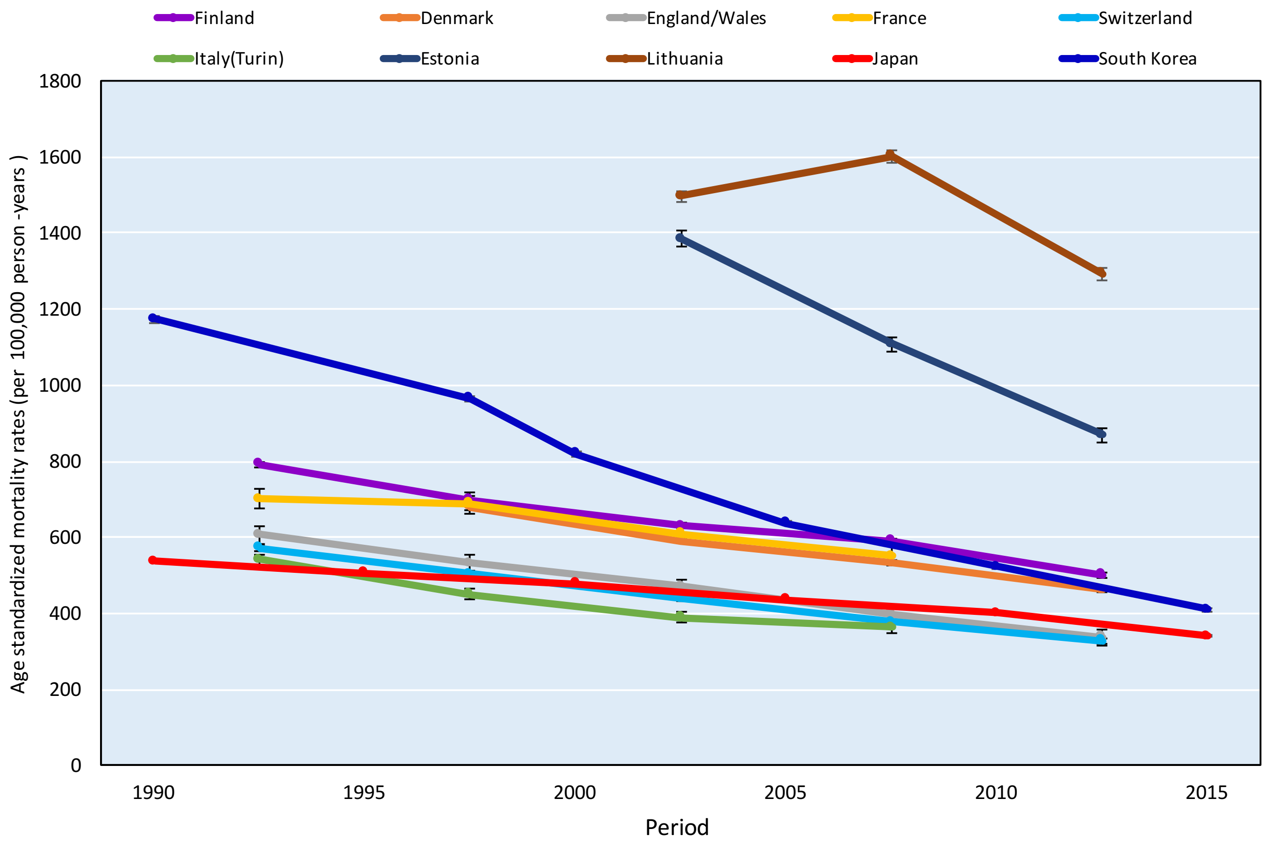

Appendix Figure 1-1. Trends in age-standardized all-cause mortality rate among the whole male population aged 35-64 years

Appendix 2 – Definition of occupational class

We categorized occupational class into five categories: upper non-manual workers (e.g. professionals, managers), lower non-manual workers (e.g. clerical, service, sales workers), manual workers (e.g. craft and related trades workers, semi-skilled and unskilled manual workers), farmers and self-employed. These occupational classes were defined following the Erikson-Goldthorpe-Portocarero (EGP) scheme which was specifically developed for international comparisons. In some countries (Finland, France Switzerland, and Italy(Turin)), data were pre-harmonized by applying the EGP scheme to national data on mortality by occupation before supplying the harmonized data to the coordinating center. For other countries (Denmark, England, Estonia, Lithuania, Japan, and South Korea), detailed data on mortality by occupation that were not yet classified according to the EGP scheme were sent to the coordinating center and post-harmonized there. Occupations in European countries were originally classified according to the International Standard Classification of Occupations (ISCO), whereas occupations in Japan and South Korea were originally classified according to the Japanese Standard Occupational Classification (JSOC) and the Korean Standard Classification of Occupations (KSCO), respectively.

Appendix Table 2-1 shows the definitions of the occupational classes used in our analysis according to ISCO, JSOC and KSCO. Appendix Table 2-2 provides the distribution of occupational classes by level of education in each of the participating countries. Appendix Table 2-3 shows percentages of self-employed by occupational class from the population census in Japan and South Korea. Because self-employed was lacking in Japan, and South Korea (and Estonia), we conducted sensitivity analysis using an indirect estimation of mortality among the self-employed in Japan and South Korea (using Appendix Table 2-3). The sensitivity analysis is presented in Appendix 5. We need to interpret with caution, especially comparison between European countries and Japan, South Korea because Japanese Standard Occupational Classification (JSOC: https://unstats.un.org/unsd/cr/ctryreg/ctrydetail.asp?id=1155) and Korean Standard Classification of Occupations (KSCO: https://unstats.un.org/unsd/cr/ctryreg/ctrydetail.asp?id=1160) do not directly correspondent to International Standard Classification of Occupations (ISCO: http://www.ilo.org/public/english/bureau/stat/isco/).

Appendix Table 2-1. Definitions of occupational class

Classification in this study	European countries: International Standard Classification of Occupations (ISCO)	Japan: Japanese Standard Occupational Classification (JSOC)	South Korea: Korean Standard Classification of Occupations (KSCO)	Correspondence to the Erikson-Goldthorpe-Portocarero (EGP) scheme
Upper non-manual workers	1. Legislators, senior officials and managers	(A) Administrative and managerial workers	KSCO1: legislators, senior officials and managers	I: Higher-grade professionals, administrators and officials; managers in large industrial establishments; large proprietors
	2. Professionals	(B) Professional and engineering workers	KSCO2: professionals	
	3. Technicians and associate professionals		KSCO3: technicians and associate professionals	II: Lower-grade professionals, administrators and officials; higher-grade technicians; managers in small business and industrial establishments; supervisors of non-manual employees
Lower non-manual workers	4. Clerks	(C) Clerical workers	KSCO4: clerks	III: Routine non-manual employees in administration and commerce; sales personnel; other rank-and-file service workers
	5. Service workers and shop and market sales workers	(D) Sales workers	KSCO5: service workers and sale workers	
		(E) Service workers		
Manual workers	7. Craft and related trades workers	(H) Manufacturing process workers	KSCO7: craft and related trades workers	V/VI: Lower-grade technicians; supervisors of manual workers; skilled manual workers
	8. Plant and machine operators and assemblers	(I) Transport and machine operating workers	KSCO8: plant and machine operators and assemblers	
		(J) Construction and mining workers		
	9. Elementary occupations	(K) Carrying, cleaning, packaging, and related workers	KSCO9: elementary occupations	VIIa: Semi- and unskilled manual workers (not in agriculture)
Farmers	6. Skilled agricultural and fishery workers	(G) Agriculture forestry and fishery workers	KSCO6: agricultural, forestry and fishery workers	IVc: Farmers and smallholders; self-employed fishermen<br>VIIb: Agricultural workers
Self-employed	Self-employed with employees, self-employed no employees (employment status)	-	-	IVa: Small proprietors; artisans, etc., with employees<br>IVb: Small proprietors, artisans, etc., without employees

Appendix Table 2-2. Percentages of educational level by occupational class (%, population census)

	1990-1999			2000-2009			2010-2014		
Educational level	Low (ISCES 0-2)	Middle (ISCED 3-4)	High (ISCED 5-6)	Low (ISCES 0-2)	Middle (ISCED 3-4)	High (ISCED 5-6)	Low (ISCES 0-2)	Middle (ISCED 3-4)	High (ISCED 5-6)
Finland	1990 (men aged 35-64)			2000 (men aged 35-64)			2010 (men aged 35-64)		
Upper non-manual	9	12	79	7	13	81	5	14	81
Lower non-manual	30	30	40	20	33	47	14	37	48
Manual	59	40	2	42	54	4	32	62	6
Farmers	64	31	5	46	46	8	32	55	13
Self-employed	48	31	20	35	40	25	26	46	28
Denmark				2000 (men aged 35-64)			2010 (men aged 35-64)		
Upper non-manual	-	-	-	8	34	58	6	32	62
Lower non-manual	-	-	-	23	59	18	21	61	18
Manual	-	-	-	34	58	7	29	62	9
Farmers	-	-	-	32	60	8	20	74	6
Self-employed	-	-	-	26	54	20	20	56	25
England/Wales	1991 (men aged 35-64)			2001 (men aged 35-64)			2011 (men aged 35-64)		
Upper non-manual	18	-	82	25	29	46	13	22	65
Lower non-manual	66	-	34	45	37	18	30	39	32
Manual	97	-	3	77	18	5	59	30	11
Farmers	-	-	-	-	-	-	-	-	-
Self-employed	-	-	-	66	23	11	47	32	21
France	*Data not available*			*Data not available*			*Data not available*		
Switzerland	1990 (men aged 35-64)			2000 (men aged 35-64)			2010 (men aged 35-64)		
Upper non-manual	4	49	47	3	38	60	2	35	62
Lower non-manual	11	86	3	1	93	7	1	94	5
Manual	44	56	0	34	66	0	30	70	0
Farmers	34	66	0	38	62	0	28	72	0
Self-employed	9	64	27	7	61	32	7	62	31

Appendix Table 2-2. Continued

Educational level	1990-1999 Low (ISCES 0-2)	1990-1999 Middle (ISCED 3-4)	1990-1999 High (ISCED 5-6)	2000-2009 Low (ISCES 0-2)	2000-2009 Middle (ISCED 3-4)	2000-2009 High (ISCED 5-6)	2010-2014 Low (ISCES 0-2)	2010-2014 Middle (ISCED 3-4)	2010-2014 High (ISCED 5-6)
Italy(Turin)	1991 (men aged 35-64)			2001 (men aged 35-64)					
Upper non-manual	8	45	47	12	32	56	-	-	-
Lower non-manual	36	51	12	23	66	11	-	-	-
Manual	94	5	0.5	80	20	1	-	-	-
Farmers	-	-	-	75	24	1	-	-	-
Self-employed	80	18	3	63	30	7	-	-	-
Estonia				2001 (men aged 35-64)			2011 (men aged 35-64)		
Upper non-manual	-	-	-	2	44	54	3	43	54
Lower non-manual	-	-	-	10	76	14	9	73	19
Manual	-	-	-	24	72	4	18	77	5
Farmers	-	-	-	33	60	7	23	68	9
Self-employed	-	-	-	-	-	-	-	-	-
Lithuania				2001 (men aged 35-64)			2011 (men aged 35-64)		
Upper non-manual	-	-	-	0.2	31	69	2	37	61
Lower non-manual	-	-	-	5	82	14	7	81	12
Manual	-	-	-	17	79	4	14	82	4
Farmers	-	-	-	30	66	4	14	75	11
Self-employed	-	-	-	16	62	22	6	59	35
Japan	1990 (men aged 35-64)			2000 (men aged 35-64)			2010 (men aged 35-64)		
Upper non-manual	8	35	58	4	30	66	2	22	77
Lower non-manual	15	52	33	9	48	44	4	41	56
Manual	47	46	8	32	55	13	17	61	21
Farmers	62	34	4	40	50	9	20	61	19
Self-employed	-	-	-	-	-	-	-	-	-

Appendix Table 2-2. Continued

	1990-1999			2000-2009			2010-2014		
Educational level	Low (ISCES 0-2)	Middle (ISCED 3-4)	High (ISCED 5-6)	Low (ISCES 0-2)	Middle (ISCED 3-4)	High (ISCED 5-6)	Low (ISCES 0-2)	Middle (ISCED 3-4)	High (ISCED 5-6)
South Korea	1995 (men aged 15 and over)			2005 (men aged 15 and over)					
Upper non-manual	4	30	66	2	18	80	-	-	-
Lower non-manual	15	53	32	7	38	55	-	-	-
Manual	33	58	9	21	57	22	-	-	-
Farmers	72	24	4	63	28	9	-	-	-
Self-employed	-	-	-	-	-	-	-	-	-

ISCED: International Standard Classification of Education

Appendix Table 2-3. Percentages of self-employed by occupational class (%); data from population census

	Self-employed (%)					
	1990	1995	2000	2005	2010	2015
Japan	(men aged 35-64)					
Upper non-manual	12.2	12.4	13.3	14.7	13.0	11.8
Lower non-manual	28.0	17.3	14.4	13.3	8.8	7.1
Manual	20.0	16.8	16.2	15.9	13.1	11.9
Farmers	80.1	84.9	81.6	80.7	74.5	69.7
Self-employed	-	-	-	-	-	-
South Korea	(men aged 15 and over)					
Upper non-manual	5.5	5.8	28.0	11.5	22.2	20.1
Lower non-manual	25.5	29.2	37.2	23.7	32.6	28.2
Manual	12.2	15.5	23.7	20.9	26.7	26.0
Farmers	94.6	93.8	94.6	93.1	92.1	93.4
Self-employed	-	-	-	-	-	-

Appendix 3 – A procedure for adjusting estimates of occupational mortality differences for the exclusion of economically inactive men

The last occupation is unknown of economically inactive men in most datasets. When these men are excluded from the calculation of mortality by occupational class, this may lead to bias because economically inactive men tend to have higher mortality than economically active men, and because men in lower occupational classes have a greater likelihood of being economically inactive than men in higher occupational classes. Therefore, we applied the procedure originally developed by Kunst et.al[1,2] to account for the exclusion of inactive men when computing occupational class differences in mortality. This algorithm was also applied by Toch-Marquardt et.al[3] on data that are also partly included in our analysis.

This algorithm aims to compute a "correction factor" that represents the extent to which the mortality rate among active men in each occupational class would have to be raised in order to correct for the exclusion of economically inactive men from that class. For occupational class z, the formula for the correction factor is:

$$1 + P_z^{inactive} * \left(RR_z^{inactive/active} - 1\right) \qquad \text{(A-3.1)}$$

with $P_z^{inactive}$ being the proportion of inactive men by occupational class, and $RR_z^{inactive/active}$ being the mortality rate ratio of inactive compared to active men by occupational class.

These proportions and relative risks by occupational class are not available in most mortality dataset, but can be estimated using information from other datasets and some simplifying assumptions.

1. The proportion of inactive men by occupational class was estimated as follows:

$$P_z^{inactive} = \mathrm{M_z} * P_{all}^{inactive} \qquad \text{(A-3.2)}$$

This formula assumes that the proportion of inactive men in occupational class z is equal to some multiplication factor M times the national average proportion of inactive men. The latter is known in all mortality datasets, whereas the multiplication factor for each occupational class was computed from EuroStat,[4] the Japanese Population Census,[5] and the South Korean social survey.[6] Appendix Table 3-1 shows the multiplication factor ($\mathrm{M_z}$) by occupational class in all countries and time-periods. Here, the multiplication factor ($\mathrm{M_z}$) means that the proportion of inactive men in occupational class z is equal to $\mathrm{M_z}$ times the national average proportion of inactive men. Appendix Table 3-2 shows percentages of occupational class unknown ($P_{all}^{inactive}$).

2. Under the simplifying assumption that there is no interaction between occupational class and activity status, the mortality rate ratio of inactive compared to active men for each occupational class can be estimated as follows:

$$RR_z^{inactive/active} = RR_{all}^{inactive/active} \qquad \text{(A-3.3)}$$

The (age-adjusted) mortality rate ration for inactive as compared to active men in the whole population could be computed in each mortality dataset. Substituting (A-3.2) and (A-3.3) into formula (A-3.1) then gives:

$$1 + \mathrm{M_z} * P_{all}^{inactive} * \left(RR_{all}^{inactive/active} - 1\right) \qquad \text{(A-3.4)}$$

The observed mortality rates in each occupational class were multiplied by the correction factor as estimated in formula (A-3.4) to derive mortality rates adjusted for the exclusion of economically inactive men.

Formula (A-3.4) can be extended to a formula for the comparison of relative inequality (class y to class z), as follows:

$$\frac{1+\mathrm{M_z}*P_{all}^{inactive}*\left(RR_{all}^{inactive/active}-1\right)}{1+\mathrm{M_y}*P_{all}^{inactive}*\left(RR_{all}^{inactive/active}-1\right)} \qquad \text{(A-3.5)}$$

Formula (A-3.5) was used as a correction factor for mortality rate ratios.

Appendix Table 3-1. Multiplication factor (M_z)* for the proportion of economically inactive men by occupational class (z)

	Period	Upper non-manual	Lower non-manual	Manual	Farmers	Self-employed
Denmark	1995-1999	0.70	0.98	1.38	1.50	0.10
	2000-2004	0.73	1.17	1.27	2.20	0.09
	2005-2009	0.67	1.02	1.39	2.10	0.11
	2010-2014	0.65	0.85	1.41	2.58	0.10
France	1990-1994	0.76	1.59	1.35	0.50	0.21
	1995-1999	0.75	1.61	1.33	0.51	0.21
	2000-2004	0.73	1.41	1.37	0.53	0.21
	2005-2009	0.73	1.42	1.35	0.55	0.21
Switzerland	1990-1994	0.80	1.47	1.83	0.50	0.16
	1995-1999	0.79	1.45	1.89	0.51	0.16
	2000-2004	0.82	1.50	1.59	0.58	0.19
	2005-2009	0.81	1.50	1.63	0.59	0.18
	2010-2014	0.84	1.37	1.47	0.62	0.19
Estonia	2000-2004	0.68	1.20	1.29	0.19	-
	2005-2009	0.65	1.15	1.33	0.18	-
	2010-2014	0.62	1.04	1.34	0.19	-
Lithuania	2000-2004	0.79	1.00	1.26	0.27	0.27
	2005-2009	0.78	0.94	1.29	0.25	0.25
	2010-2014	0.68	0.83	1.29	0.26	0.26
Japan	1990	0.66	0.70	1.21	1.84	-
	1995	0.67	0.72	1.27	1.62	-
	2000	0.75	0.68	1.26	1.93	-
	2005	0.86	0.71	1.20	1.75	-
	2010	0.85	0.69	1.24	1.62	-
	2015	0.85	0.78	1.21	1.55	-
South Korea	1990	1.01	0.92	1.22	0.78	-
	1997	0.65	1.46	1.12	0.46	-
	2000	0.66	1.31	1.10	0.62	-
	2005	0.70	1.19	1.06	0.82	-
	2010	0.76	1.15	0.99	1.12	-
	2015	0.82	1.10	0.96	1.50	-

*The proportion of economically inactive men by occupational class, estimated from EuroStat, the Japanese Population Census, and the South Korean social survey.

§ Assumption; relative ratio of the proportion of economically inactive for farmers and self-employed was assumed to equal to lower non-manual.

Occupational classification was defined by ISCO-88 (International Standard Classification of Occupations) in European countries aged 15-74;
Upper non-manual worker: Managers, Professionals, Technicians and associate professionals
Lower non-manual worker: Clerical support workers, Service and sales workers
Manual worker: Craft and related trades workers, Plant and machine operators and assemblers, Elementary occupations
Farmers: Skilled agricultural, forestry and fishery workers, Self-employed: None

Appendix Table 3-2. Percentages of occupational class unknown among men aged 35-64 (%)

	1990-1994	1995-1999	2000-2004	2005-2009	2010-2014	2015
Finland	1.3	1.5	3.0	3.5	4.2	-
Denmark*	-	22.6	20.4	32.2	22.0	-
England/Wales	3.1	3.2	3.4	3.6	4.8	-
France*	4.7	3.9	6.5	5.6	-	-
Switzerland*	6.2	4.9	22.5	19.8	21.3	-
Italy(Turin)	4.4	3.4	7.6	7.6	-	-
Estonia*	-	-	30.4	25.8	30.8	-
Lithuania*	-	-	39.5	35.5	36.8	-
Japan*	8.3	9.2	11.9	17.1	22.7	22.3
South Korea*	12.6	11.3	16.6	17.6	16.0	14.9

*Correction method was applied to these countries.

Reference

1. Kunst AE, Groenhof F, Mackenbach JP. Mortality by occupational class among men 30-64 years in 11 European countries. EU Working Group on Socioeconomic Inequalities in Health. *Soc Sci Med.* 1998;46(11):1459-1476.
2. Kunst AE, Groenhof F, Mackenbach JP, Health EW. Occupational class and cause specific mortality in middle aged men in 11 European countries: comparison of population based studies. EU Working Group on Socioeconomic Inequalities in Health. *BMJ.* 1998;316(7145):1636-1642.
3. Toch-Marquardt M, Menvielle G, Eikemo TA, et al. Occupational class inequalities in all-cause and cause-specific mortality among middle-aged men in 14 European populations during the early 2000s. *PLoS One.* 2014;9(9):e108072.
4. Eurostat. Previous occupations of the unemployed, by sex (1000). 2017; http://ec.europa.eu/eurostat/web/products-datasets/product?code=lfsa_ugpis. [accessed 10 December 2017].
5. Portal site of official statistics of Japan. National census. 2017; https://www.e-stat.go.jp/stat-search/database?page=1&toukei=00200521&result_page=1. [accessed 10 December 2017]
6. KOrea Statistical Information Service. Working persons by city&province/gender/type of work/occupation (15 years and over). 2017; http://kosis.kr/statHtml/statHtml.do?orgId=101&tblId=DT_1BA9504&conn_path=I3 [accessed 5 March 2018].

Appendix 4 – Age-standardized mortality rate (ASMR) by occupational class

This appendix describes the original data we analyzed. Appendix Table 4-1 to 4-10 show detailed age-standardized mortality rate by country. Appendix Table 4-11 shows age-standardized mortality rate difference calculated by original data shown in Figure 1. The mortality differences in 8 Western European countries were clear and showed social gradients; that is, upper non-manual workers had the lowest mortality and manual workers had the highest mortality. Meanwhile, the trends of mortality by occupational class were different in Japan and South Korea.

Figures shown in this appendix are follows;
-Appendix Figure 4-1. Male age-standardized mortality rate and population distribution by occupational class among 8 European countries, Japan, and South Korea by study period (after applying correction factor)
- Appendix Figure 4-2. Trends in male all-cause mortality rate by occupational class (after applying correction factor)
- Appendix Figure 4-3. Changes in broad cause-specific contribution to inequality (%) among five occupational class estimated by average inter-group difference (AID absolute version)
- Appendix Figure 4-4. Age-standardized mortality rate aged 35-64 men and contribution of cause-specific death by countries (before applying correction factor)

Appendix Table 4-1. Age-standardized all-cause and cause-specific mortality by occupational class, men aged 35-64 in Finland (per 100,000 person-years)

Finland		1990-1994	1995-1999	2000-2004	2005-2009	2010-2014	Change*			1990-1994	1995-1999	2000-2004	2005-2009	2010-2014	Change*
**All-cause**								**Cause-specific death**							
All-cause	All population	791	699	631	592	502	-290	Ischemic heart disease	All population	215	161	125	100	79	-136
	Upper non-manual	456	380	342	308	248	-208	(I20-I25)	Upper non-manual	117	82	63	48	37	-80
	Lower non-manual	618	536	498	455	416	-202		Lower non-manual	173	124	101	79	62	-111
	Manual	989	901	812	767	640	-349		Manual	265	206	163	131	107	-157
	Farmers	683	622	549	519	469	-215		Farmers	204	163	114	102	81	-124
	Self-employed	663	564	505	495	402	-262		Self-employed	174	137	101	80	55	-119
**Broad cause-specific death**															
All cancer	All population	172	156	143	131	117	-55	Cerebrovascular disease	All population	47	38	32	25	20	-27
(C00-D48)	Upper non-manual	130	108	97	90	77	-53	(I60-I69)	Upper non-manual	30	19	19	12	10	-20
	Lower non-manual	149	137	128	116	110	-39		Lower non-manual	37	31	28	19	15	-21
	Manual	200	186	173	156	140	-60		Manual	57	46	39	33	25	-32
	Farmers	147	134	124	103	87	-61		Farmers	40	44	29	28	24	-16
	Self-employed	153	142	120	126	109	-44		Self-employed	41	35	28	23	17	-24
Cardiovascular disease	All population	316	245	204	176	146	-170	Smoking-related causes	All population	64	54	46	42	37	-27
(I00-I99)	Upper non-manual	179	126	106	88	70	-109	(C32-34, J40-44, J47)	Upper non-manual	28	17	19	17	13	-15
	Lower non-manual	250	188	164	134	113	-137		Lower non-manual	45	39	29	31	30	-15
	Manual	388	312	263	230	193	-195		Manual	87	78	67	60	55	-32
	Farmers	297	252	190	178	144	-153		Farmers	46	37	36	28	22	-24
	Self-employed	263	205	170	144	110	-153		Self-employed	47	37	34	38	21	-25
External causes	All population	176	155	143	138	103	-73	Suicide	All population	63	52	43	39	31	-32
(V01-Y98)	Upper non-manual	80	74	71	63	44	-36	(X60-X84, Y87.0)	Upper non-manual	30	28	22	20	12	-18
	Lower non-manual	121	108	98	96	81	-40		Lower non-manual	47	39	32	31	26	-20
	Manual	239	213	190	183	132	-107		Manual	81	67	55	49	40	-41
	Farmers	133	126	117	126	118	-16		Farmers	55	49	43	48	44	-11
	Self-employed	149	115	115	114	86	-63		Self-employed	62	39	34	29	28	-34
Other causes	All population	128	143	142	147	136	8	Road traffic accidents	All population	14	13	12	9	8	-7
	Upper non-manual	67	71	69	68	57	-10	(V01-V89, Y85)	Upper non-manual	9	6	7	5	4	-5
	Lower non-manual	99	103	107	110	113	15		Lower non-manual	10	10	7	6	5	-5
	Manual	162	190	187	197	176	14		Manual	18	16	14	11	9	-9
	Farmers	106	110	118	112	121	15		Farmers	12	13	16	14	14	3
	Self-employed	98	102	99	112	97	-1		Self-employed	16	15	15	10	9	-7

*Absolute mortality changes between 1990-1994 and 2010-2014.

Appendix Table 4-2. Age-standardized all-cause and cause-specific mortality by occupational class, men aged 35-64 in Denmark (per 100,000 person-years)

Denmark		1995-1999	2000-2004	2005-2009	2010-2014	Change*			1995-1999	2000-2004	2005-2009	2010-2014	Change*
**All-cause**							**Cause-specific death**						
All-cause	All population	679	591	533	462	-217	Ischemic heart disease	All population	100	66	46	36	-64
	Upper non-manual	444	380	329	276	-168	(I20-I25)	Upper non-manual	66	39	30	20	-46
	Lower non-manual	700	686	588	513	-187		Lower non-manual	84	84	47	38	-46
	Manual	883	743	773	635	-248		Manual	122	76	64	50	-73
	Farmers	747	868	1192	974	227		Farmers	112	100	129	46	-66
	Self-employed	379	324	263	202	-177		Self-employed	64	40	23	18	-46
**Broad cause-specific death**													
All cancer	All population	218	198	178	159	-59	Cerebrovascular disease	All population	30	28	22	16	-14
(C00-D48)	Upper non-manual	163	143	130	114	-48	(I60-I69)	Upper non-manual	18	20	13	9	-9
	Lower non-manual	206	213	189	168	-38		Lower non-manual	26	28	24	15	-11
	Manual	265	229	225	195	-71		Manual	36	32	32	22	-14
	Farmers	223	245	316	215	-8		Farmers	14	44	11	73	59
	Self-employed	146	140	116	91	-55		Self-employed	17	15	10	6	-11
Cardiovascular disease	All population	174	139	106	82	-92	Smoking-related causes	All population	82	69	62	55	-27
(I00-I99)	Upper non-manual	116	91	70	49	-66	(C32-34, J40-44, J47)	Upper non-manual	44	37	31	27	-18
	Lower non-manual	162	168	106	85	-77		Lower non-manual	78	71	67	57	-20
	Manual	208	160	148	109	-99		Manual	114	87	99	83	-31
	Farmers	166	162	220	164	-2		Farmers	86	103	128	43	-42
	Self-employed	105	81	52	38	-67		Self-employed	37	36	28	19	-18
External causes	All population	69	60	56	51	-19	Suicide	All population	29	25	21	23	-6
(V01-Y98)	Upper non-manual	40	34	29	31	-8	(X60-X84, Y87.0)	Upper non-manual	19	16	12	16	-3
	Lower non-manual	74	73	57	53	-21		Lower non-manual	32	31	23	25	-7
	Manual	105	89	96	78	-26		Manual	38	34	32	33	-5
	Farmers	198	57	77	123	-75		Farmers	63	18	21	50	-13
	Self-employed	44	38	34	25	-18		Self-employed	22	19	17	13	-8
Other causes	All population	218	194	193	170	-48	Road traffic accidents	All population	11	10	8	6	-5
	Upper non-manual	126	111	100	81	-45	(V01-V89, Y85)	Upper non-manual	6	6	5	3	-3
	Lower non-manual	258	231	236	207	-51		Lower non-manual	10	10	7	6	-4
	Manual	305	265	305	253	-52		Manual	16	14	13	8	-7
	Farmers	160	404	578	472	312		Farmers	35	12	19	6	-28
	Self-employed	84	66	62	48	-37		Self-employed	10	7	6	3	-6

*Absolute mortality changes between 1995-1999 and 2010-2014.

Appendix Table 4-3. Age-standardized all-cause and cause-specific mortality by occupational class, men aged 35-64 in England/Wales (per 100,000 person-years)

England/Wales		1990-1994	1995-1999	2000-2004	2005-2009	2010-2014	Change*			1990-1994	1995-1999	2000-2004	2005-2009	2010-2014	Change*
**All-cause**								**Cause-specific death**							
All-cause	All population	606	532	471	398	337	-134	Ischemic heart disease	All population	192	150	103	79	58	-45
	Upper non-manual	465	329	342	276	240	-101	(I20-I25)	Upper non-manual	144	90	70	55	45	-25
	Lower non-manual	480	421	376	391	290	-85		Lower non-manual	138	118	81	96	52	-30
	Manual	671	600	585	504	407	-177		Manual	226	171	136	104	63	-73
	Farmers	-	-	-	-	-	-		Farmers	-	-	-	-	-	-
	Self-employed	-	-	424	361	289	-135		Self-employed	-	-	85	67	46	-39
**Broad cause-specific death**															
All cancer	All population	214	190	171	147	129	-42	Cerebrovascular disease	All population	31	24	20	15	11	-9
(C00-D48)	Upper non-manual	180	137	153	123	107	-46	(I60-I69)	Upper non-manual	18	22	13	8	6	-7
	Lower non-manual	182	170	123	159	102	-20		Lower non-manual	26	16	4	16	15	11
	Manual	233	205	197	167	147	-50		Manual	34	30	22	17	14	-8
	Farmers	-	-	-	-	-	-		Farmers	-	-	-	-	-	-
	Self-employed	-	-	157	148	128	-29		Self-employed	-	-	23	20	5	-17
Cardiovascular disease	All population	257	203	151	118	89	-62	Smoking-related causes	All population	88	65	63	42	42	-21
(I00-I99)	Upper non-manual	199	136	102	79	60	-42	(C32-34, J40-44, J47)	Upper non-manual	41	18	40	26	26	-14
	Lower non-manual	197	157	114	119	92	-22		Lower non-manual	59	40	38	46	25	-12
	Manual	292	231	191	154	106	-85		Manual	106	84	82	59	55	-27
	Farmers	-	-	-	-	-	-		Farmers	-	-	-	-	-	-
	Self-employed	-	-	138	112	62	-76		Self-employed	-	-	63	39	39	-24
External causes	All population	31	37	32	30	27	-5	Suicide	All population	12	11	10	12	10	0
(V01-Y98)	Upper non-manual	20	12	22	21	19	-3	(X60-X84, Y87.0)	Upper non-manual	6	6	7	8	11	4
	Lower non-manual	24	29	14	34	15	1		Lower non-manual	8	9	0	16	5	5
	Manual	36	43	40	43	32	-9		Manual	16	13	11	17	10	-1
	Farmers	-	-	-	-	-	-		Farmers	-	-	-	-	-	-
	Self-employed	-	-	45	24	40	-5		Self-employed	-	-	16	20	11	-5
Other causes	All population	105	102	118	103	92	-26	Road traffic accidents	All population	5	7	7	4	2	-5
	Upper non-manual	65	44	64	53	54	-11	(V01-V89, Y85)	Upper non-manual	5	0	5	2	2	-3
	Lower non-manual	77	65	124	79	81	-43		Lower non-manual	6	8	6	12	0	-6
	Manual	110	121	157	140	123	-34		Manual	4	8	8	6	2	-6
	Farmers	-	-	-	-	-	-		Farmers	-	-	-	-	-	-
	Self-employed	-	-	83	77	58	-25		Self-employed	-	-	10	1	2	-8

*Absolute mortality changes between 2000-2004 and 2010-2014.

Appendix Table 4-4. Age-standardized all-cause and cause-specific mortality by occupational class, men aged 35-64 in France (per 100,000 person-years)

France		1990-1994	1995-1999	2000-2004	2005-2009	Change*			1990-1994	1995-1999	2000-2004	2005-2009	Change*
**All-cause**							**Cause-specific death**						
All-cause	All population	700	689	610	552	-149	Ischemic heart disease	All population	59	51	44	40	-19
	Upper non-manual	457	438	379	386	-71	(I20-I25)	Upper non-manual	50	31	32	24	-26
	Lower non-manual	943	838	822	694	-248		Lower non-manual	65	59	38	43	-23
	Manual	980	983	912	772	-208		Manual	69	76	65	54	-15
	Farmers	500	575	422	410	-91		Farmers	45	38	31	55	10
	Self-employed	436	484	411	441	5		Self-employed	51	30	33	45	-6
**Broad cause-specific death**													
All cancer	All population	300	305	265	242	-58	Cerebrovascular disease	All population	25	25	18	17	-8
(C00-D48)	Upper non-manual	217	206	184	192	-25	(I60-I69)	Upper non-manual	10	7	13	8	-2
	Lower non-manual	390	367	330	272	-118		Lower non-manual	25	22	17	19	-7
	Manual	416	435	375	333	-83		Manual	37	39	26	28	-9
	Farmers	191	244	188	151	-39		Farmers	27	39	16	14	-12
	Self-employed	173	183	169	187	14		Self-employed	22	18	5	14	-8
Cardiovascular disease	All population	134	123	99	95	-38	Smoking-related causes	All population	43	31	35	42	-2
(I00-I99)	Upper non-manual	86	69	62	63	-23	(C32-34, J40-44, J47)	Upper non-manual	68	20	23	26	-42
	Lower non-manual	169	170	114	109	-59		Lower non-manual	129	29	60	43	-86
	Manual	172	175	148	132	-40		Manual	150	44	40	60	-91
	Farmers	106	109	72	97	-10		Farmers	51	32	32	44	-7
	Self-employed	112	86	73	93	-19		Self-employed	48	26	39	32	-16
External causes	All population	98	89	76	76	-22	Suicide	All population	101	107	84	86	-16
(V01-Y98)	Upper non-manual	64	57	48	49	-15	(X60-X84, Y87.0)	Upper non-manual	31	65	50	60	29
	Lower non-manual	122	94	115	91	-31		Lower non-manual	62	148	70	92	29
	Manual	134	118	99	106	-28		Manual	58	160	142	135	77
	Farmers	84	93	66	71	-13		Farmers	24	79	57	44	20
	Self-employed	83	95	73	67	-16		Self-employed	41	49	53	49	7
Other causes	All population	169	172	170	138	-30	Road traffic accidents	All population	20	21	10	9	-10
	Upper non-manual	90	106	85	83	-7	(V01-V89, Y85)	Upper non-manual	15	19	4	4	-12
	Lower non-manual	261	208	263	222	-40		Lower non-manual	14	13	12	8	-6
	Manual	258	255	291	201	-57		Manual	28	22	17	14	-14
	Farmers	119	129	96	91	-28		Farmers	25	35	11	16	-9
	Self-employed	67	121	97	95	27		Self-employed	0	21	5	15	15

*Absolute mortality changes between 1990-1994 and 2005-2009.

Appendix Table 4-5. Age-standardized all-cause and cause-specific mortality by occupational class, men aged 35-64 in Switzerland (per 100,000 person-years)

Switzerland		1990-1994	1995-1999	2000-2004	2005-2009	2010-2014	Change*			1990-1994	1995-1999	2000-2004	2005-2009	2010-2014	Change*
**All-cause**								**Cause-specific death**							
All-cause	All population	571	504	438	378	327	-244	Ischemic heart disease	All population	93	74	54	46	35	-58
	Upper non-manual	421	383	304	279	240	-181	(I20-I25)	Upper non-manual	73	60	41	34	25	-48
	Lower non-manual	693	625	566	467	422	-270		Lower non-manual	110	94	64	50	40	-70
	Manual	894	791	711	616	524	-369		Manual	128	101	78	69	54	-74
	Farmers	445	441	382	357	335	-110		Farmers	57	59	43	42	46	-11
	Self-employed	529	488	359	332	293	-236		Self-employed	88	72	46	47	34	-54
**Broad cause-specific death**															
All cancer	All population	205	183	162	145	123	-81	Cerebrovascular disease	All population	15	14	11	8	7	-8
(C00-D48)	Upper non-manual	158	146	118	116	99	-58	(I60-I69)	Upper non-manual	10	9	6	6	4	-6
	Lower non-manual	236	219	202	166	139	-97		Lower non-manual	18	17	13	9	11	-7
	Manual	296	274	248	217	187	-109		Manual	24	24	18	15	10	-13
	Farmers	166	157	134	133	110	-56		Farmers	10	10	9	8	6	-4
	Self-employed	195	175	137	130	119	-76		Self-employed	10	13	10	7	6	-5
Cardiovascular disease	All population	164	130	101	84	70	-93	Smoking-related causes	All population	77	65	54	46	38	-39
(I00-I99)	Upper non-manual	121	101	74	62	50	-72	(C32-34, J40-44, J47)	Upper non-manual	48	43	32	29	25	-23
	Lower non-manual	197	162	117	97	85	-111		Lower non-manual	93	83	72	60	47	-46
	Manual	247	192	154	135	112	-135		Manual	134	118	98	83	74	-61
	Farmers	116	121	78	81	89	-28		Farmers	59	63	42	50	30	-29
	Self-employed	146	127	89	79	65	-82		Self-employed	68	57	39	40	35	-34
External causes	All population	85	71	63	59	47	-38	Suicide	All population	43	40	35	30	25	-18
(V01-Y98)	Upper non-manual	62	54	43	43	35	-27	(X60-X84, Y87.0)	Upper non-manual	32	32	24	22	19	-14
	Lower non-manual	97	81	76	73	51	-46		Lower non-manual	50	46	43	44	29	-21
	Manual	140	98	98	95	70	-70		Manual	67	52	52	47	37	-31
	Farmers	93	90	91	75	70	-23		Farmers	47	40	38	33	37	-9
	Self-employed	88	76	65	52	41	-47		Self-employed	52	49	45	29	24	-28
Other causes	All population	118	120	111	91	87	-31	Road traffic accidents	All population	13	10	9	7	5	-8
	Upper non-manual	79	81	68	59	56	-24	(V01-V89, Y85)	Upper non-manual	9	7	6	5	4	-5
	Lower non-manual	163	163	171	131	147	-16		Lower non-manual	16	14	9	6	3	-13
	Manual	211	226	211	167	155	-55		Manual	20	12	14	10	7	-13
	Farmers	69	73	78	68	66	-3		Farmers	18	12	19	14	10	-8
	Self-employed	100	110	68	72	68	-32		Self-employed	15	13	10	10	6	-9

*Absolute mortality changes between 1990-1994 and 2010-2014.

Appendix Table 4-6. Age-standardized all-cause and cause-specific mortality by occupational class, men aged 35-64 in Italy (Turin) (per 100,000 person-years)

Italy(Turin)		1990-1994	1995-1999	2000-2004	2005-2009	Change*			1990-1994	1995-1999	2000-2004	2005-2009	Change*
**All-cause**							**Cause-specific death**						
All-cause	All population	542	450	390	363	-178	Ischemic heart disease	All population	75	59	46	41	-33
	Upper non-manual	341	290	235	236	-104	(I20-I25)	Upper non-manual	53	39	27	32	-20
	Lower non-manual	479	374	317	275	-204		Lower non-manual	73	50	49	35	-38
	Manual	566	494	442	392	-174		Manual	72	65	51	40	-31
	Farmers	-	-	521	657	-		Farmers	-	-	94	146	-
	Self-employed	507	461	349	378	-129		Self-employed	77	61	42	38	-39
**Broad cause-specific death**													
All cancer	All population	233	201	183	168	-65	Cerebrovascular disease	All population	30	18	15	13	-18
(C00-D48)	Upper non-manual	147	148	118	126	-21	(I60-I69)	Upper non-manual	22	12	9	11	-12
	Lower non-manual	200	179	155	130	-70		Lower non-manual	31	7	11	12	-19
	Manual	257	220	223	191	-66		Manual	31	20	16	13	-19
	Farmers	-	-	181	292	-		Farmers	-	-	45	0	-
	Self-employed	222	205	154	186	-36		Self-employed	24	19	12	12	-12
Cardiovascular disease	All population	156	120	95	81	-75	Smoking-related causes	All population	94	81	67	57	-37
(I00-I99)	Upper non-manual	105	73	62	58	-48	(C32-34, J40-44, J47)	Upper non-manual	54	56	37	9	-45
	Lower non-manual	159	92	85	64	-96		Lower non-manual	76	60	53	11	-65
	Manual	152	133	103	82	-70		Manual	103	92	81	12	-91
	Farmers	-	-	245	365	-		Farmers	-	-	136	0	-
	Self-employed	142	125	90	79	-63		Self-employed	96	80	63	10	-87
External causes	All population	42	32	34	31	-12	Suicide	All population	15	11	14	12	-3
(V01-Y98)	Upper non-manual	32	17	26	17	-15	(X60-X84, Y87.0)	Upper non-manual	13	9	11	30	17
	Lower non-manual	36	30	23	22	-14		Lower non-manual	15	12	12	39	24
	Manual	42	33	30	34	-8		Manual	14	10	15	73	59
	Farmers	-	-	54	0	-		Farmers	-	-	54	73	-
	Self-employed	41	40	44	32	-9		Self-employed	15	13	12	64	49
Other causes	All population	111	98	79	83	-27	Road traffic accidents	All population	14	9	9	7	-6
	Upper non-manual	57	52	30	36	-21	(V01-V89, Y85)	Upper non-manual	12	5	8	2	-10
	Lower non-manual	85	74	55	60	-25		Lower non-manual	14	9	5	4	-10
	Manual	114	108	87	84	-30		Manual	13	8	7	11	-2
	Farmers	-	-	42	0	-		Farmers	-	-	0	0	-
	Self-employed	102	91	62	81	-21		Self-employed	11	17	17	11	0

*Absolute mortality changes between 1990-1994 and 2005-2010.

Appendix Table 4-7. Age-standardized all-cause and cause-specific mortality by occupational class, men aged 35-64 in Estonia (per 100,000 person-years)

Estonia		2000-2004	2005-2009	2010-2014	Change*			2000-2004	2005-2009	2010-2014	Change*
**All-cause**						**Cause-specific death**					
All-cause	All population	1385	1107	870	-515	Ischemic heart disease	All population	279	186	127	-153
	Upper non-manual	763	612	470	-294	(I20-I25)	Upper non-manual	169	110	68	-102
	Lower non-manual	1280	1266	846	-434		Lower non-manual	230	213	125	-106
	Manual	1639	1397	1064	-575		Manual	304	225	158	-146
	Farmers	1295	1152	806	-489		Farmers	240	191	83	-157
	Self-employed	-	-	-	-		Self-employed	-	-	-	-
**Broad cause-specific death**											
All cancer	All population	282	248	221	-61	Cerebrovascular disease	All population	102	54	32	-71
(C00-D48)	Upper non-manual	178	144	140	-39	(I60-I69)	Upper non-manual	58	26	21	-37
	Lower non-manual	281	273	228	-53		Lower non-manual	102	75	10	-91
	Manual	314	314	255	-60		Manual	112	69	32	-80
	Farmers	283	253	190	-93		Farmers	80	52	25	-56
	Self-employed	-	-	-	-		Self-employed	-	-	-	-
Cardiovascular disease	All population	516	401	295	-222	Smoking-related causes	All population	62	41	33	-30
(I00-I99)	Upper non-manual	296	229	151	-145	(C32-34, J40-44, J47)	Upper non-manual	46	34	29	-17
	Lower non-manual	454	469	293	-162		Lower non-manual	88	88	77	-11
	Manual	586	502	362	-224		Manual	124	118	92	-32
	Farmers	425	384	210	-215		Farmers	119	103	55	-63
	Self-employed	-	-	-	-		Self-employed	-	-	-	-
External causes	All population	322	217	153	-169	Suicide	All population	107	87	72	-35
(V01-Y98)	Upper non-manual	165	109	68	-96	(X60-X84, Y87.0)	Upper non-manual	40	23	18	-22
	Lower non-manual	272	235	155	-118		Lower non-manual	54	31	28	-26
	Manual	404	280	211	-192		Manual	76	53	43	-33
	Farmers	356	263	211	-146		Farmers	65	52	45	-20
	Self-employed	-	-	-	-		Self-employed	-	-	-	-
Other causes	All population	264	241	201	-63	Road traffic accidents	All population	29	18	10	-19
	Upper non-manual	124	129	111	-14	(V01-V89, Y85)	Upper non-manual	18	14	7	-12
	Lower non-manual	272	290	171	-101		Lower non-manual	36	22	6	-30
	Manual	335	300	237	-98		Manual	35	20	13	-21
	Farmers	232	252	196	-35		Farmers	33	18	10	-24
	Self-employed	-	-	-	-		Self-employed	-	-	-	-

*Absolute mortality changes between 2000-2004 and 2010-2015.

Appendix Table 4-8. Age-standardized all cause and cause-specific mortality by occupational class, men aged 35-64 in Lithuania (per 100,000 person-years)

Lithuania		2000-2004	2005-2009	2010-2014	Change*			2000-2004	2005-2009	2010-2014	Change*
**All-cause**						**Cause-specific death**					
All-cause	All population	1496	1602	1292	-204	Ischemic heart disease	All population	310	317	264	-46
	Upper non-manual	745	806	714	-32	(I20-I25)	Upper non-manual	172	177	165	-7
	Lower non-manual	1127	1104	1066	-61		Lower non-manual	236	245	232	-4
	Manual	1599	1845	1714	115		Manual	329	368	307	-21
	Farmers	1705	1972	1050	-655		Farmers	331	328	213	-118
	Self-employed	1369	1494	620	-748		Self-employed	264	282	132	-133
**Broad cause-specific death**											
All cancer	All population	305	305	272	-33	Cerebrovascular disease	All population	81	85	74	-7
(C00-D48)	Upper non-manual	182	176	166	-16	(I60-I69)	Upper non-manual	39	49	38	-1
	Lower non-manual	253	263	253	0		Lower non-manual	81	80	64	-17
	Manual	334	347	333	0		Manual	90	99	92	2
	Farmers	320	370	245	-75		Farmers	77	82	38	-39
	Self-employed	264	306	138	-126		Self-employed	59	74	38	-21
Cardiovascular disease	All population	513	551	445	-68	Smoking-related causes	All population	126	124	100	-25
(I00-I99)	Upper non-manual	281	316	269	-12	(C32-34, J40-44, J47)	Upper non-manual	43	37	38	-4
	Lower non-manual	404	412	385	-19		Lower non-manual	75	79	83	8
	Manual	548	638	540	-8		Manual	138	145	142	4
	Farmers	519	571	324	-195		Farmers	167	210	108	-59
	Self-employed	434	490	217	-217		Self-employed	113	126	31	-82
External causes	All population	293	256	197	-96	Suicide	All population	118	83	82	-35
(V01-Y98)	Upper non-manual	132	106	92	-40	(X60-X84, Y87.0)	Upper non-manual	47	29	34	-13
	Lower non-manual	202	146	150	-52		Lower non-manual	74	51	55	-19
	Manual	327	305	290	-36		Manual	128	95	123	-5
	Farmers	413	417	233	-180		Farmers	218	167	120	-97
	Self-employed	299	239	114	-185		Self-employed	130	89	57	-74
Other causes	All population	385	491	378	-8	Road traffic accidents	All population	49	39	21	-28
	Upper non-manual	151	208	187	36	(V01-V89, Y85)	Upper non-manual	26	18	12	-14
	Lower non-manual	269	283	278	9		Lower non-manual	38	23	22	-16
	Manual	390	555	550	160		Manual	53	43	30	-23
	Farmers	453	613	247	-205		Farmers	72	76	21	-51
	Self-employed	372	459	152	-221		Self-employed	56	41	11	-45

*Absolute mortality changes between 2000-2004 and 2010-2015.

Appendix Table 4-9. Age-standardized all-cause and cause-specific mortality by occupational class, men aged 35-64 in Japan (per 100,000 person-years)

Japan		1990	1995	2000	2005	2010	2015	Change*			1990	1995	2000	2005	2010	2015	Change*
**All-cause**									**Cause-specific death**								
All-cause	All population	537	505	478	436	401	340	-165	Ischemic heart disease	All population	NA	37	35	35	33	28	-9
	Upper non-manual	414	408	642	616	498	357	-51	(I20-I25)	Upper non-manual	NA	34	53	51	47	35	1
	Lower non-manual	568	502	412	347	292	258	-244		Lower non-manual	NA	44	36	35	31	24	-20
	Manual	453	452	352	335	356	338	-114		Manual	NA	35	31	32	34	32	-4
	Farmers	1057	1082	1297	1193	1122	916	-166		Farmers	NA	47	46	53	52	43	-4
	Self-employed	-	-	-	-	-	-	-		Self-employed	-	-	-	-	-	-	-
**Broad cause-specific death**																	
All cancer	All population	NA	204	184	161	143	123	-82	Cerebrovascular disease	All population	NA	52	44	38	33	27	-25
(C00-C97)	Upper non-manual	NA	181	267	255	198	141	-39	(I60-I69)	Upper non-manual	NA	43	63	54	42	31	-12
	Lower non-manual	NA	209	157	128	105	94	-115		Lower non-manual	NA	57	44	38	31	23	-34
	Manual	NA	154	112	100	111	112	-42		Manual	NA	50	36	36	37	31	-20
	Farmers	NA	326	355	319	296	229	-97		Farmers	NA	80	74	59	59	51	-29
	Self-employed	-	-	-	-	-	-	-		Self-employed	-	-	-	-	-	-	-
Cardiovascular disease	All population	NA	119	108	104	96	82	-37	Smoking-related causes	All population	NA	34	33	33	31	28	-6
(I01-I02.0, I10-I13, I05-I09, I20-I25, I27, I30-I51, I60-I69, I71)	Upper non-manual	NA	96	142	137	111	85	-11	(C33-34, J41-44)	Upper non-manual	NA	31	50	58	47	32	1
	Lower non-manual	NA	121	95	87	74	63	-58		Lower non-manual	NA	36	32	29	27	24	-12
	Manual	NA	105	81	84	89	85	-20		Manual	NA	27	23	24	27	30	2
	Farmers	NA	228	256	246	231	203	-25		Farmers	NA	46	45	41	43	37	-9
	Self-employed	-	-	-	-	-	-	-		Self-employed	-	-	-	-	-	-	-
External causes	All population	NA	69	84	77	68	49	-21	Suicide	All population	NA	33	53	51	44	32	-1
(V01-X84)	Upper non-manual	NA	51	111	99	78	50	0	(X60-X84)	Upper non-manual	NA	29	83	75	61	40	11
	Lower non-manual	NA	61	72	60	49	36	-25		Lower non-manual	NA	40	62	51	42	29	-11
	Manual	NA	86	84	77	70	52	-34		Manual	NA	43	59	54	50	34	-9
	Farmers	NA	233	311	278	245	192	-41		Farmers	NA	92	123	130	115	99	7
	Self-employed	-	-	-	-	-	-	-		Self-employed	-	-	-	-	-	-	-
Other causes	All population	NA	112	102	94	94	87	-26	Road traffic accidents	All population	NA	16	13	10	6	5	-11
	Upper non-manual	NA	80	123	125	111	80	0	(V01-V98)	Upper non-manual	NA	10	16	12	7	5	-5
	Lower non-manual	NA	111	88	72	64	65	-45		Lower non-manual	NA	14	11	8	5	4	-11
	Manual	NA	106	76	74	86	89	-18		Manual	NA	20	14	11	8	6	-13
	Farmers	NA	295	374	350	350	292	-3		Farmers	NA	39	36	26	15	12	-27
	Self-employed	-	-	-	-	-	-	-		Self-employed	-	-	-	-	-	-	-

*Absolute mortality changes between 1995 and 2015. NA; data not available

Appendix Table 4-10. Age-standardized all-cause and cause-specific mortality by occupational class, men aged 35-64 in South Korea (per 100,000 person-years)

South Korea		1990	1997	2000	2005	2010	2015	Change*			1990	1997	2000	2005	2010	2015	Change*
**All-cause**									**Cause-specific death**								
All-cause	All population	1174	964	821	637	523	410	-555	Ischemic heart disease	All population	NA	32	38	33	26	21	-11
	Upper non-manual	360	377	384	355	479	495	118	(I20-I25)	Upper non-manual	NA	20	26	24	30	30	10
	Lower non-manual	937	1399	1132	961	751	438	-961		Lower non-manual	NA	68	64	56	41	26	-42
	Manual	1011	813	542	363	290	297	-516		Manual	NA	28	26	19	13	15	-13
	Farmers	1753	1388	1429	1143	1067	998	-390		Farmers	NA	24	41	38	29	25	1
	Self-employed	-	-	-	-	-	-	-		Self-employed	-	-	-	-	-	-	-
**Broad cause-specific death**																	
All cancer	All population	NA	311	269	222	179	138	-173	Cerebrovascular disease	All population	NA	106	85	55	33	25	-81
(C00-D48)	Upper non-manual	NA	153	156	146	190	211	58	(I60-I69)	Upper non-manual	NA	42	39	32	32	28	-14
	Lower non-manual	NA	497	415	384	283	169	-328		Lower non-manual	NA	154	112	79	45	28	-126
	Manual	NA	234	144	96	73	79	-155		Manual	NA	84	52	31	18	18	-66
	Farmers	NA	391	397	313	267	246	-145		Farmers	NA	129	124	72	47	41	-87
	Self-employed	-	-	-	-	-	-	-		Self-employed	-	-	-	-	-	-	-
Cardiovascular disease	All population	NA	191	156	110	79	65	-126	Smoking-related causes	All population	NA	69	64	51	39	30	-40
(I00-I99)	Upper non-manual	NA	76	74	65	75	80	5	(C32-34, J40-44, J47)	Upper non-manual	NA	27	28	30	45	43	16
	Lower non-manual	NA	297	235	174	118	75	-222		Lower non-manual	NA	95	85	75	49	34	-61
	Manual	NA	152	97	65	43	46	-106		Manual	NA	49	35	23	15	16	-33
	Farmers	NA	241	221	141	108	111	-130		Farmers	NA	96	103	76	60	54	-42
	Self-employed	-	-	-	-	-	-	-		Self-employed	-	-	-	-	-	-	-
External causes	All population	NA	163	135	125	116	87	-76	Suicide	All population	NA	30	32	50	57	48	17
(V01-Y98)	Upper non-manual	NA	57	56	65	98	96	38	(X60-X84, Y87.0)	Upper non-manual	NA	9	12	31	60	61	52
	Lower non-manual	NA	200	157	162	145	85	-115		Lower non-manual	NA	40	40	74	82	55	15
	Manual	NA	185	124	99	85	76	-110		Manual	NA	34	26	33	33	39	5
	Farmers	NA	266	308	303	307	246	-20		Farmers	NA	54	86	118	123	83	30
	Self-employed	-	-	-	-	-	-	-		Self-employed	-	-	-	-	-	-	-
Other causes	All population	NA	299	260	180	150	119	-181	Road traffic accidents	All population	NA	72	53	32	24	16	-57
	Upper non-manual	NA	91	97	79	116	108	17	(V01-V89, Y85)	Upper non-manual	NA	26	23	14	19	17	-9
	Lower non-manual	NA	406	326	241	205	109	-297		Lower non-manual	NA	92	63	42	28	15	-77
	Manual	NA	242	176	103	89	96	-145		Manual	NA	74	47	25	18	13	-61
	Farmers	NA	490	504	386	384	395	-95		Farmers	NA	124	121	92	72	56	-68
	Self-employed	-	-	-	-	-	-	-		Self-employed	-	-	-	-	-	-	-

*Absolute mortality changes between 1997 and 2015. NA; data not available

Appendix Table 4-11. Age-standardized mortality rate difference (RD as compared to upper non-manual worker, by countries: observation period: 2010-2014 (2005-2009*, 2015†)

	All-cause	All cancer		Cardiovascular disease		External causes		Other causes	
	RD	RD	(%‡)	RD	(%‡)	RD	(%‡)	RD	(%‡)
**Finland**									
Upper non-manual	0								
Lower non-manual	168	33	(20)	43	(25)	36	(22)	56	(33)
Manual	392	63	(16)	123	(31)	87	(22)	119	(30)
Farmers	221	10	(4)	74	(34)	73	(33)	63	(29)
Self-employed	154	32	(21)	40	(26)	42	(27)	40	(26)
**Denmark**									
Upper non-manual	0								
Lower non-manual	237	54	(23)	35	(15)	22	(9)	126	(53)
Manual	359	81	(22)	59	(17)	47	(13)	172	(48)
Farmers	698	101	(14)	115	(16)	92	(13)	391	(56)
Self-employed	-74	-23	(32)	-11	(15)	-6	(8)	-33	(45)
**England/Wales**									
Upper non-manual	0								
Lower non-manual	50	-5	(7)	32	(46)	-4	(6)	28	(40)
Manual	167	40	(24)	46	(27)	12	(7)	69	(41)
Farmers	-	-		-		-		-	
Self-employed	49	21	(43)	2	(4)	21	(44)	4	(9)
**France***									
Upper non-manual	0								
Lower non-manual	308	81	(26)	47	(15)	42	(14)	139	(45)
Manual	385	141	(37)	69	(18)	57	(15)	118	(30)
Farmers	24	-40	(39)	34	(32)	22	(21)	8	(8)
Self-employed	55	-5	(7)	30	(46)	18	(28)	11	(18)
**Switzerland**									
Upper non-manual	0								
Lower non-manual	182	40	(22)	36	(20)	16	(9)	91	(50)
Manual	284	87	(31)	62	(22)	35	(12)	99	(35)
Farmers	95	11	(12)	39	(41)	35	(37)	10	(10)
Self-employed	53	19	(37)	15	(28)	6	(12)	12	(23)

	All-cause	All cancer		Cardiovascular disease		External causes		Other causes	
	RD	RD	(%‡)	RD	(%‡)	RD	(%‡)	RD	(%‡)
**Italy(Turin)***									
Upper non-manual	0								
Lower non-manual	39	3	(9)	6	(16)	5	(14)	24	(62)
Manual	155	65	(42)	25	(16)	17	(11)	49	(31)
Farmers	421	166	(32)	307	(58)	-17	(3)	-36	(7)
Self-employed	142	60	(42)	21	(15)	15	(10)	46	(32)
**Estonia**									
Upper non-manual	0								
Lower non-manual	376	88	(23)	142	(38)	86	(23)	61	(16)
Manual	594	115	(19)	211	(35)	143	(24)	126	(21)
Farmers	337	50	(15)	59	(18)	142	(42)	86	(25)
Self-employed	-	-		-		-		-	
**Lithuania**									
Upper non-manual	0								
Lower non-manual	353	86	(25)	116	(33)	58	(16)	92	(26)
Manual	1001	167	(17)	271	(27)	199	(20)	364	(36)
Farmers	336	79	(23)	55	(16)	141	(42)	61	(18)
Self-employed	-93	-29	(21)	-52	(38)	22	(16)	-35	(25)
**Japan†**									
Upper non-manual	0								
Lower non-manual	-99	-47	(48)	-22	(23)	-14	(14)	-15	(15)
Manual	-19	-29	(75)	0	(0)	1	(3)	9	(22)
Farmers	559	88	(16)	118	(21)	142	(25)	211	(38)
Self-employed	-	-		-		-		-	
**South Korea†**									
Upper non-manual	0								
Lower non-manual	-57	-43	(71)	-5	(9)	-11	(18)	1	(2)
Manual	-199	-133	(67)	-34	(17)	-20	(10)	-12	(6)
Farmers	502	35	(7)	31	(6)	150	(30)	287	(57)
Self-employed	-	-		-		-		-	

§ Rate difference (RD) was calculated using direct method with European standard population.

‡ Percentage (%)= $|RD_{cause\text{-}specific}|/(|RD_{all\ cancer}|+|RD_{cardiovascular\ disease}|+|RD_{external\ causes}|+|RD_{other\ causes}|)$

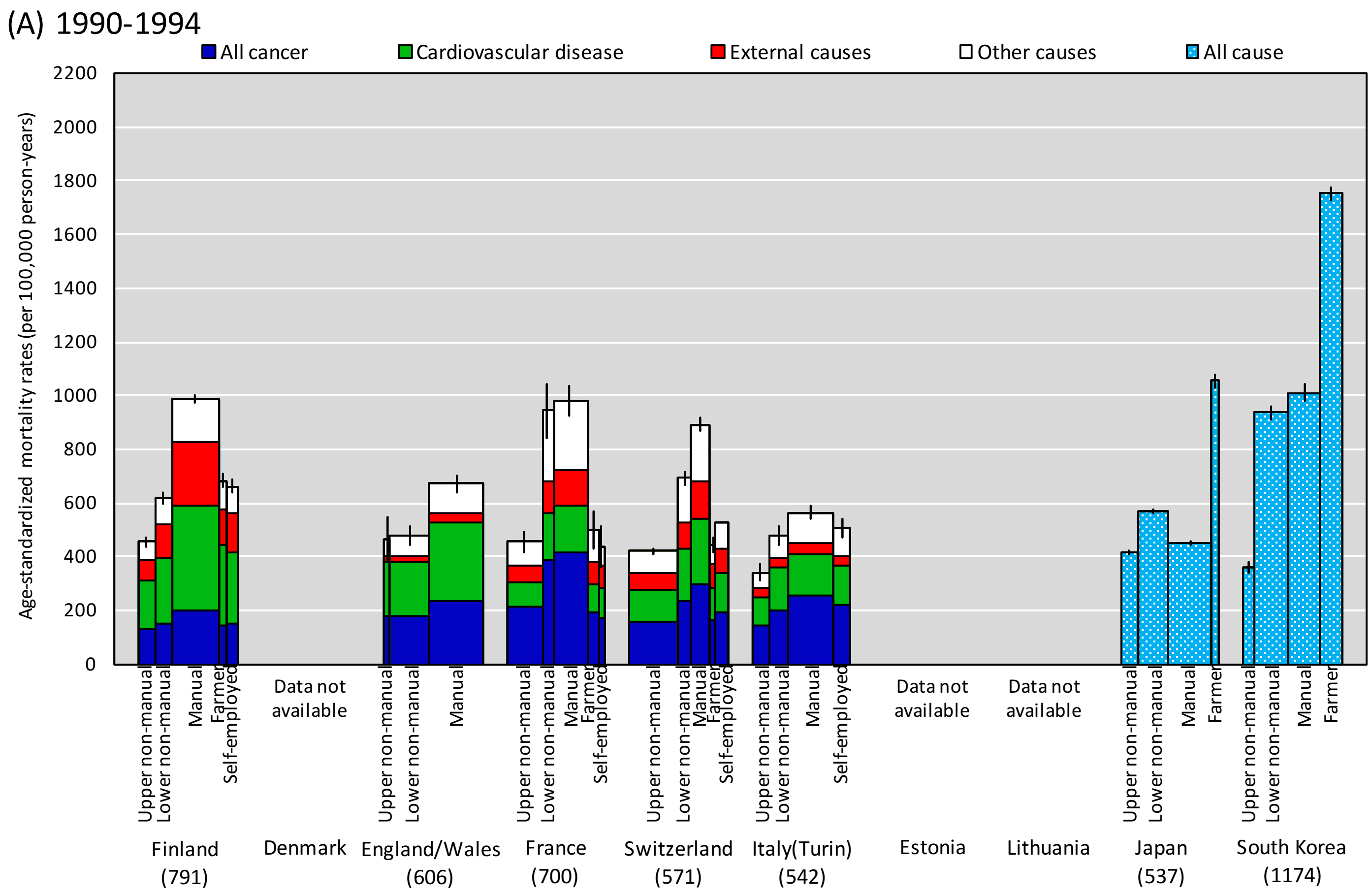

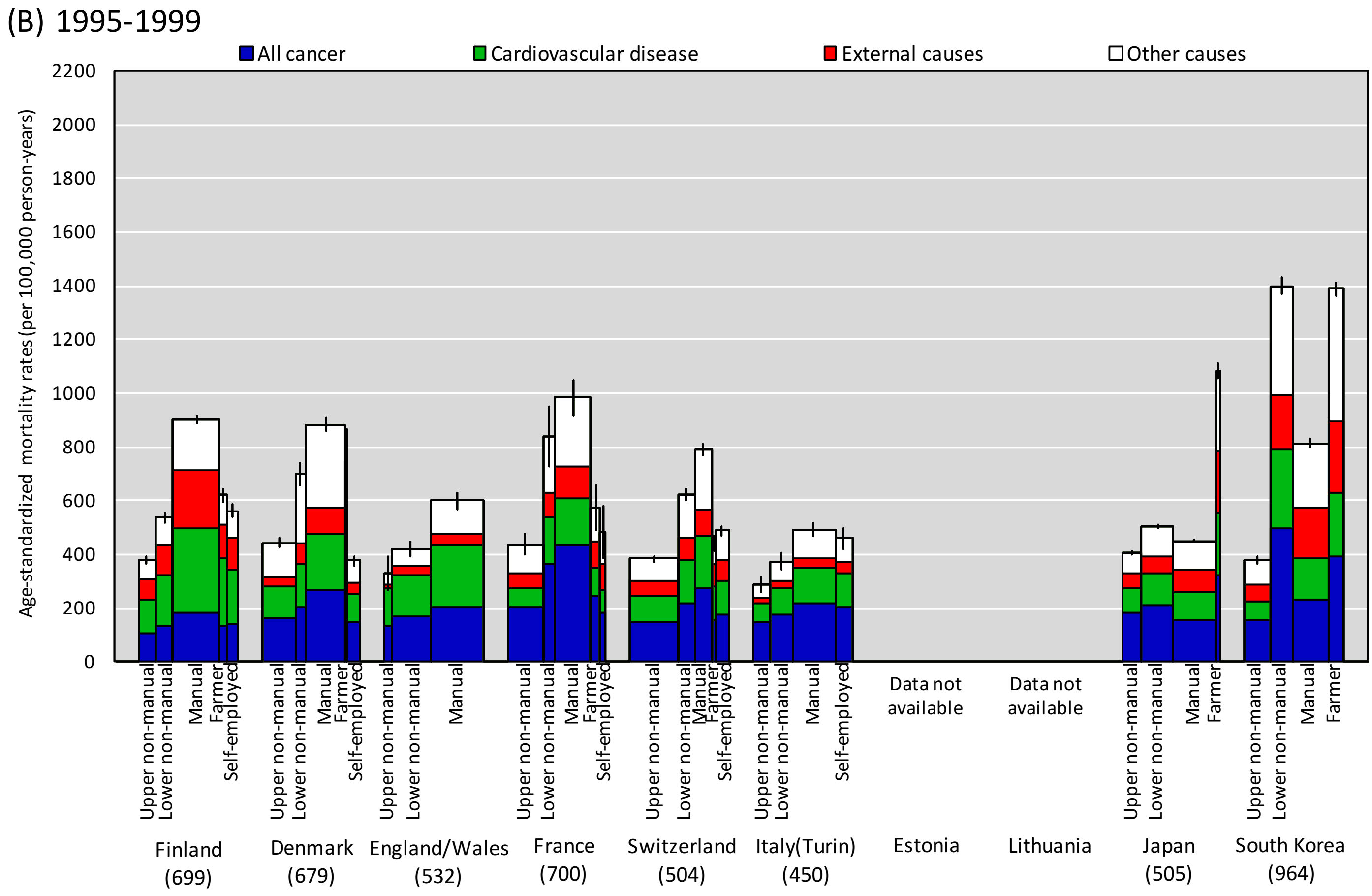

Appendix Figure 4-1. Male age-standardized mortality rate and population distribution by occupational class among 8 European countries, Japan, and South Korea by study period: number in parentheses indicate the whole population mortality rate (per 100,000 person-years): <u>after</u> applying correction factor

(C) 2000-2004

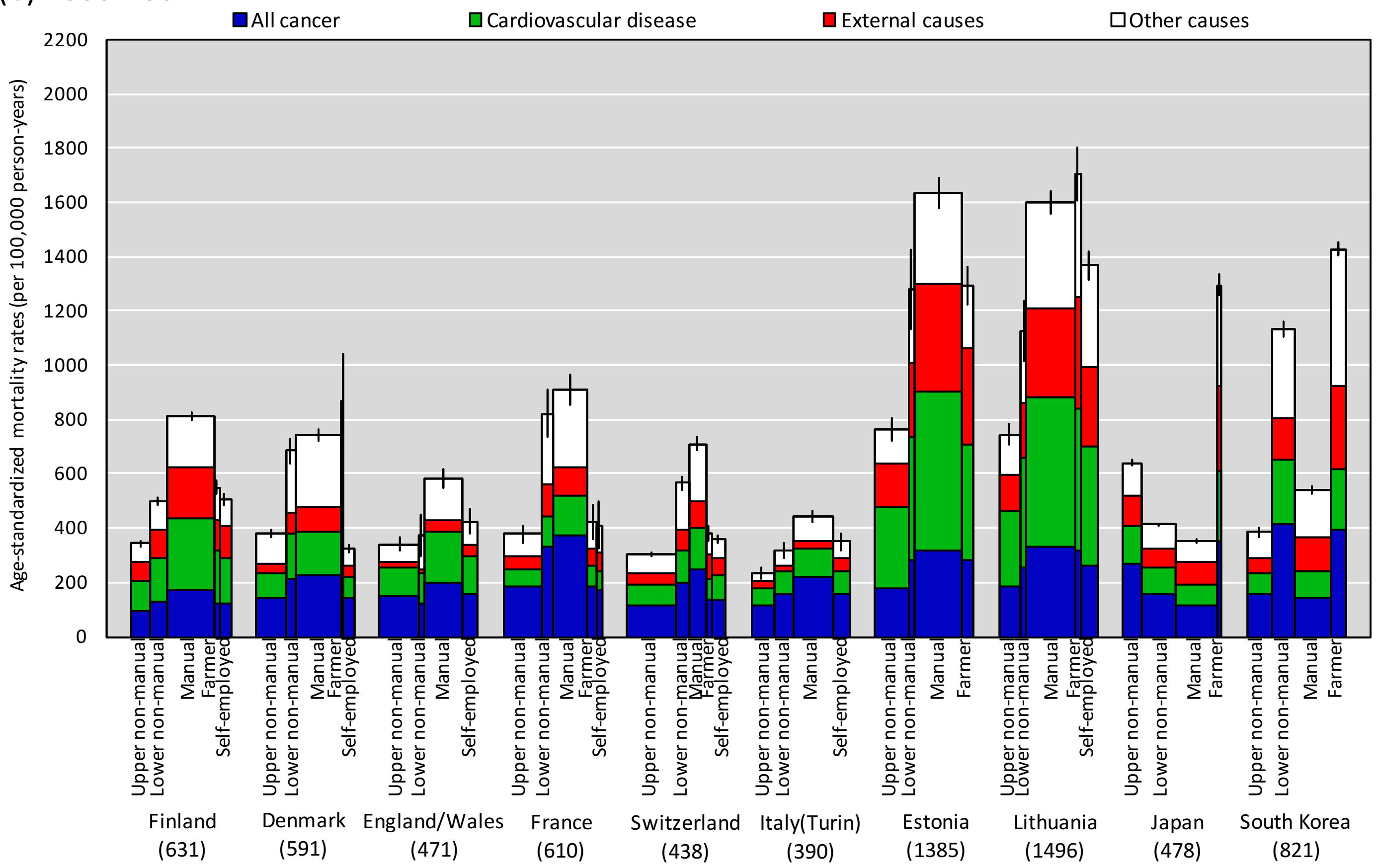

(D) 2005-2009

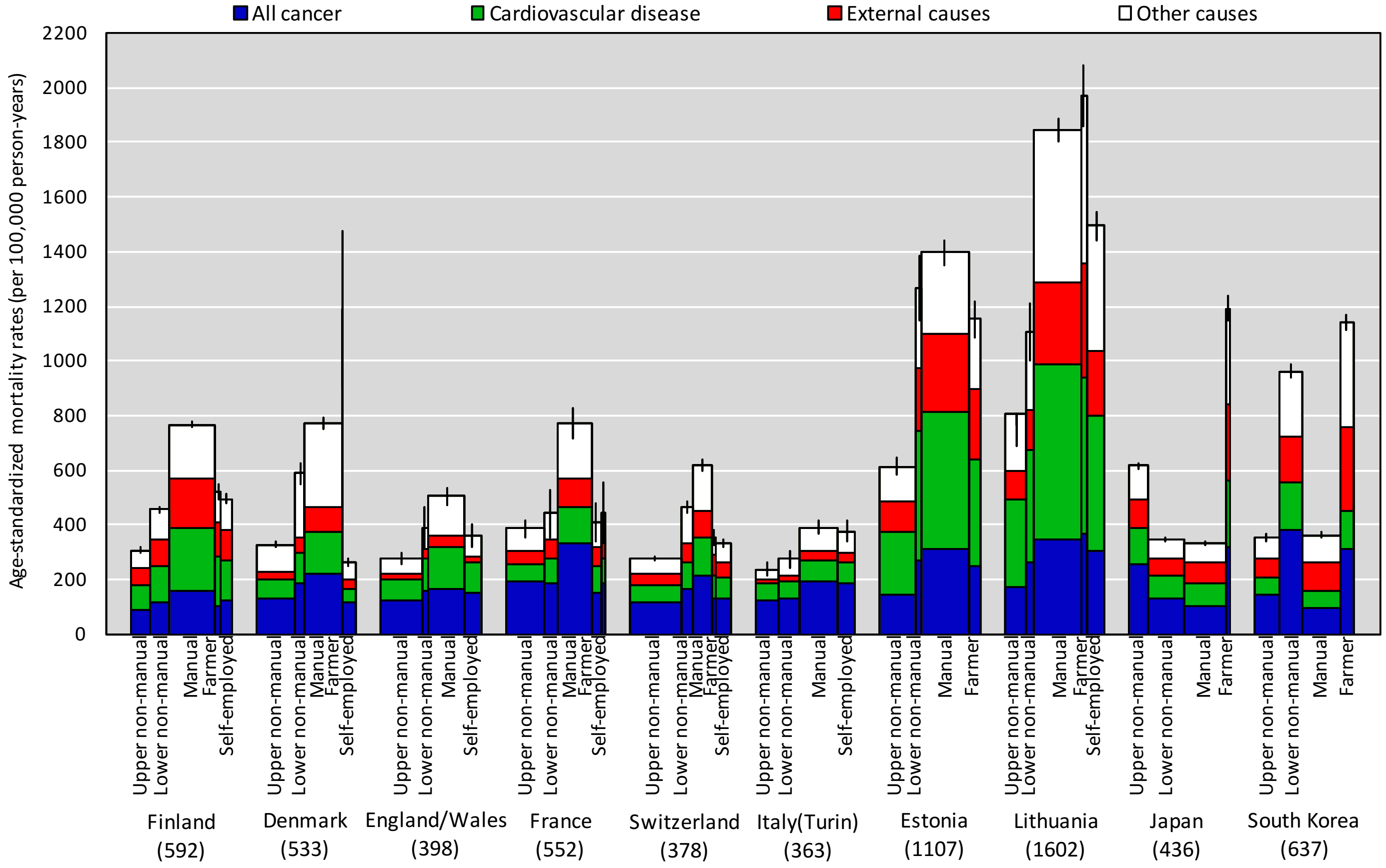

Appendix Figure 4-1. Continued

(E) 2010-2014

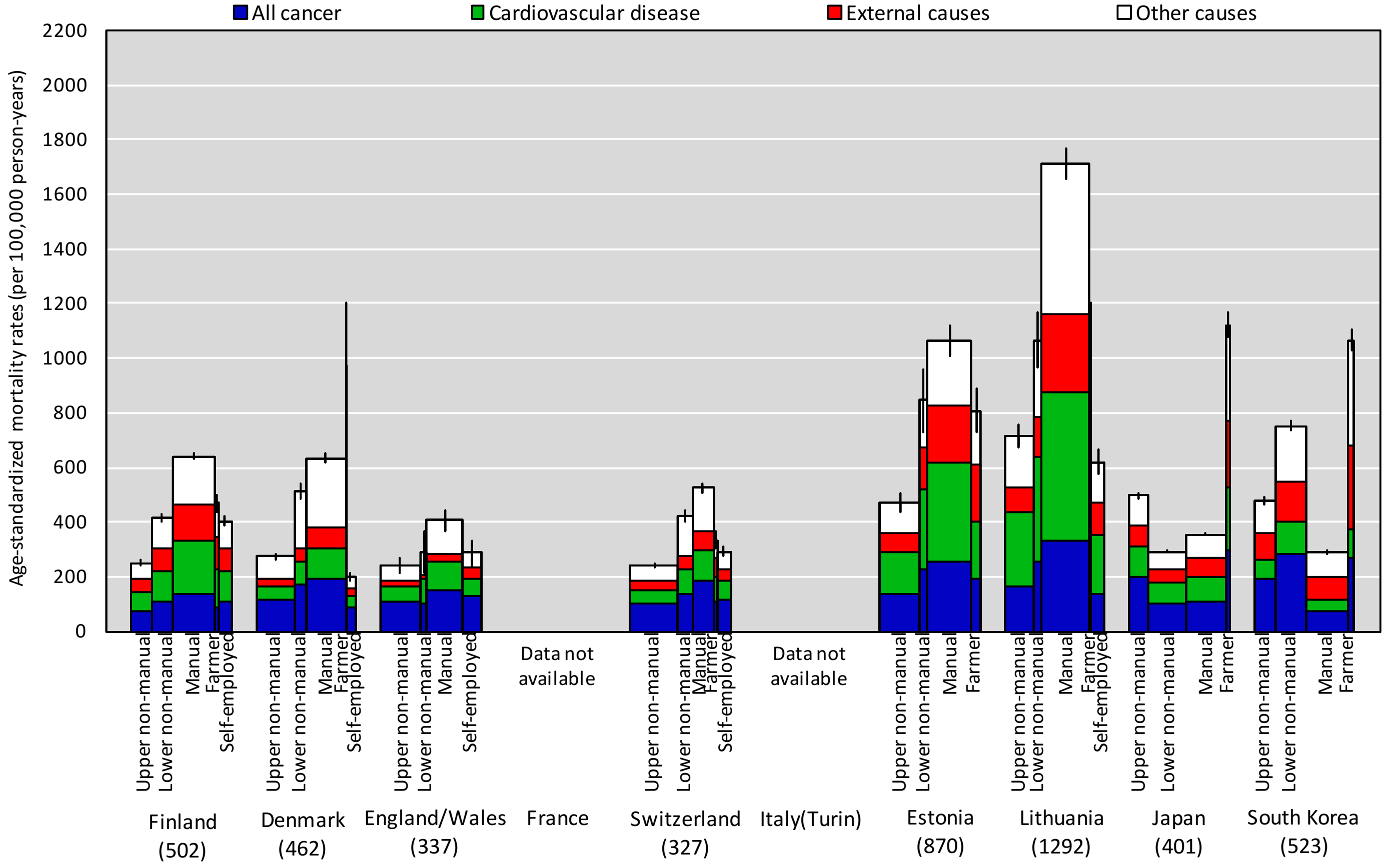

(F) 2015

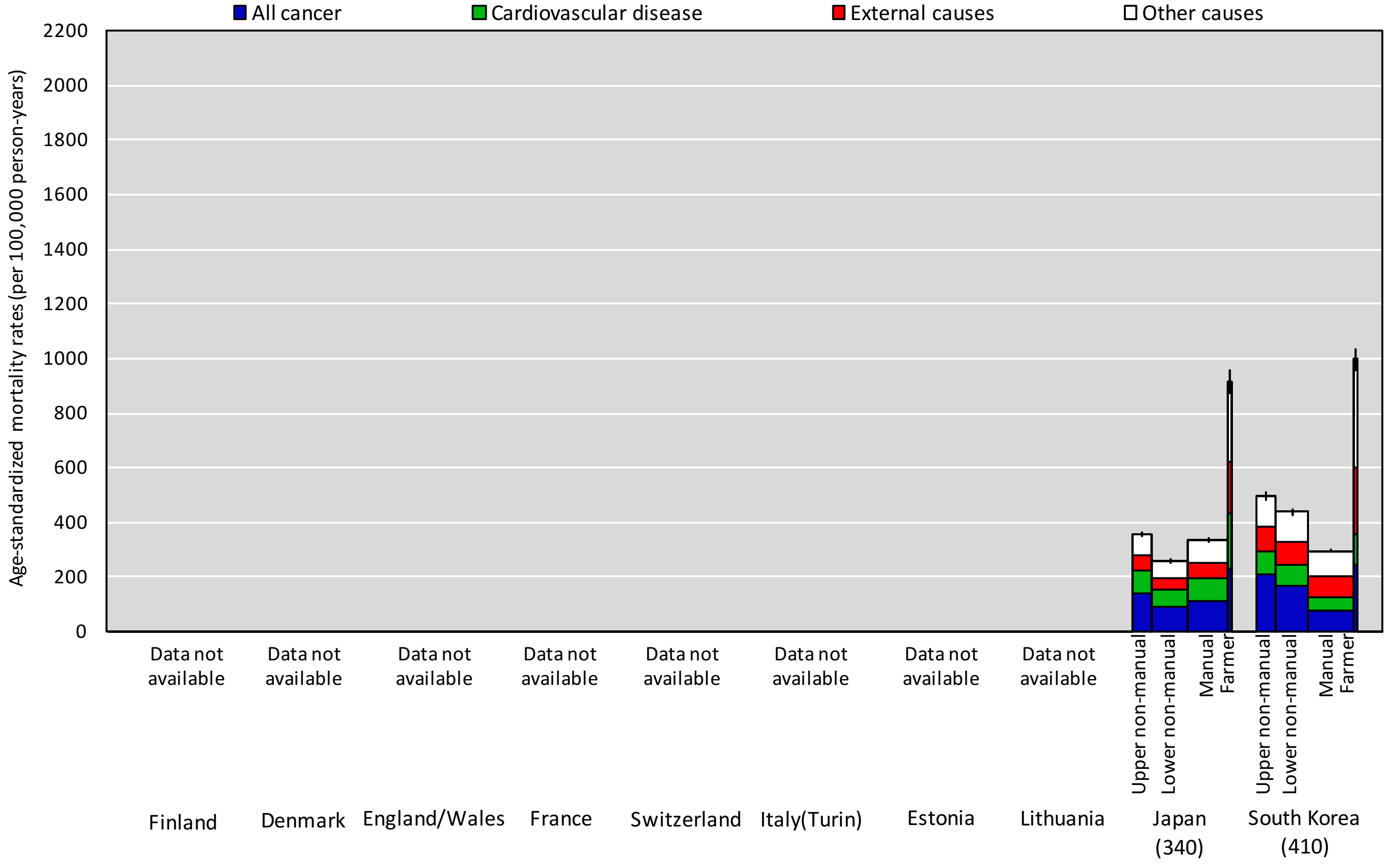

Appendix Figure 4-1. Continued

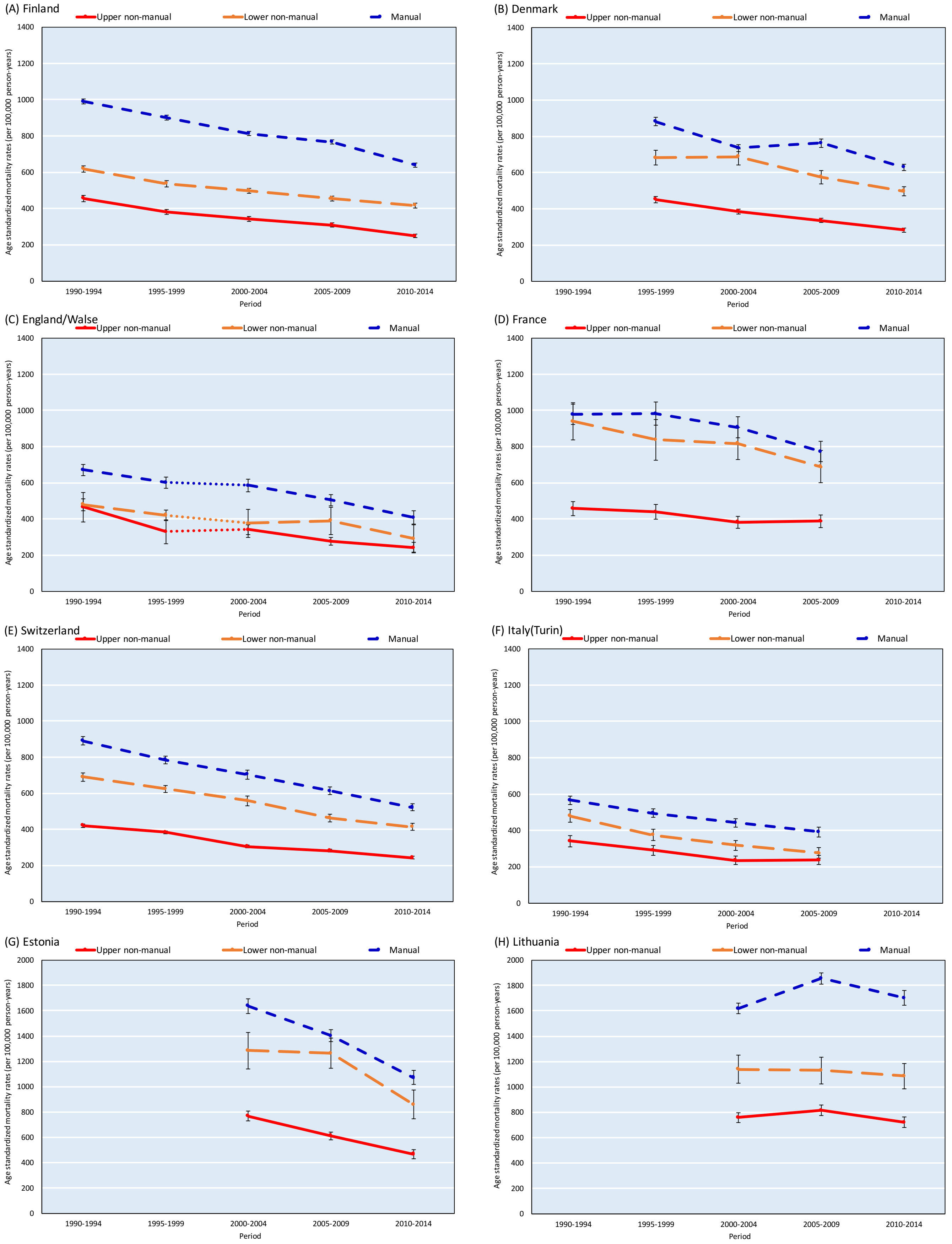

Appendix Figure 4-2. Trends in male age-standardized all-cause mortality rate by occupational class (upper non-manual, lower non-manual, and manual workers): after applying correction factor

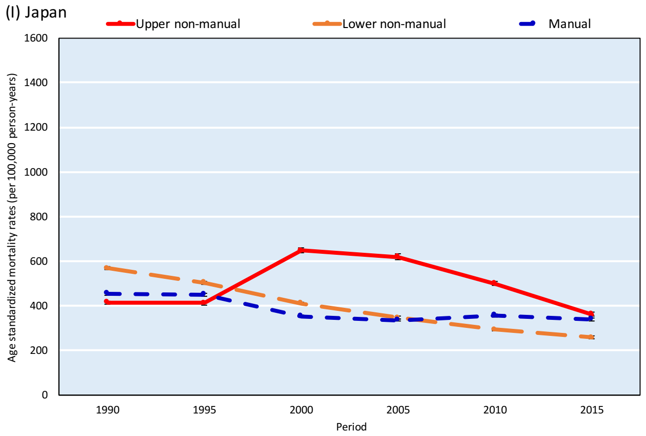

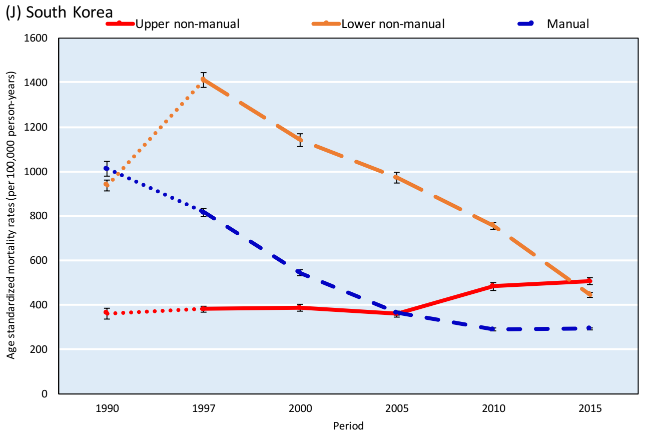

Appendix Figure 4-2. Continued

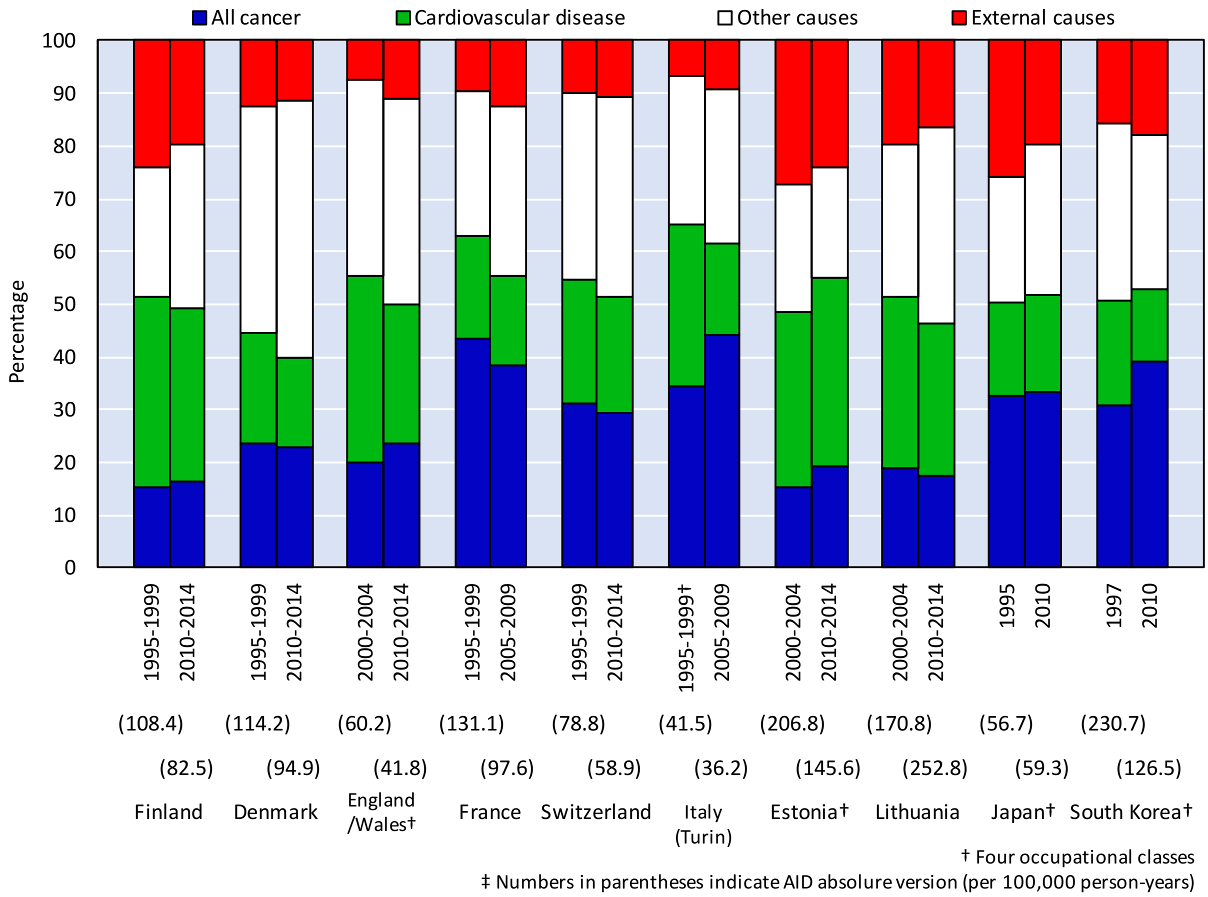

Appendix Figure 4-3. Changes in broad cause-specific contribution to inequality (%) among five occupational class estimated by average inter-group difference (AID absolute version)

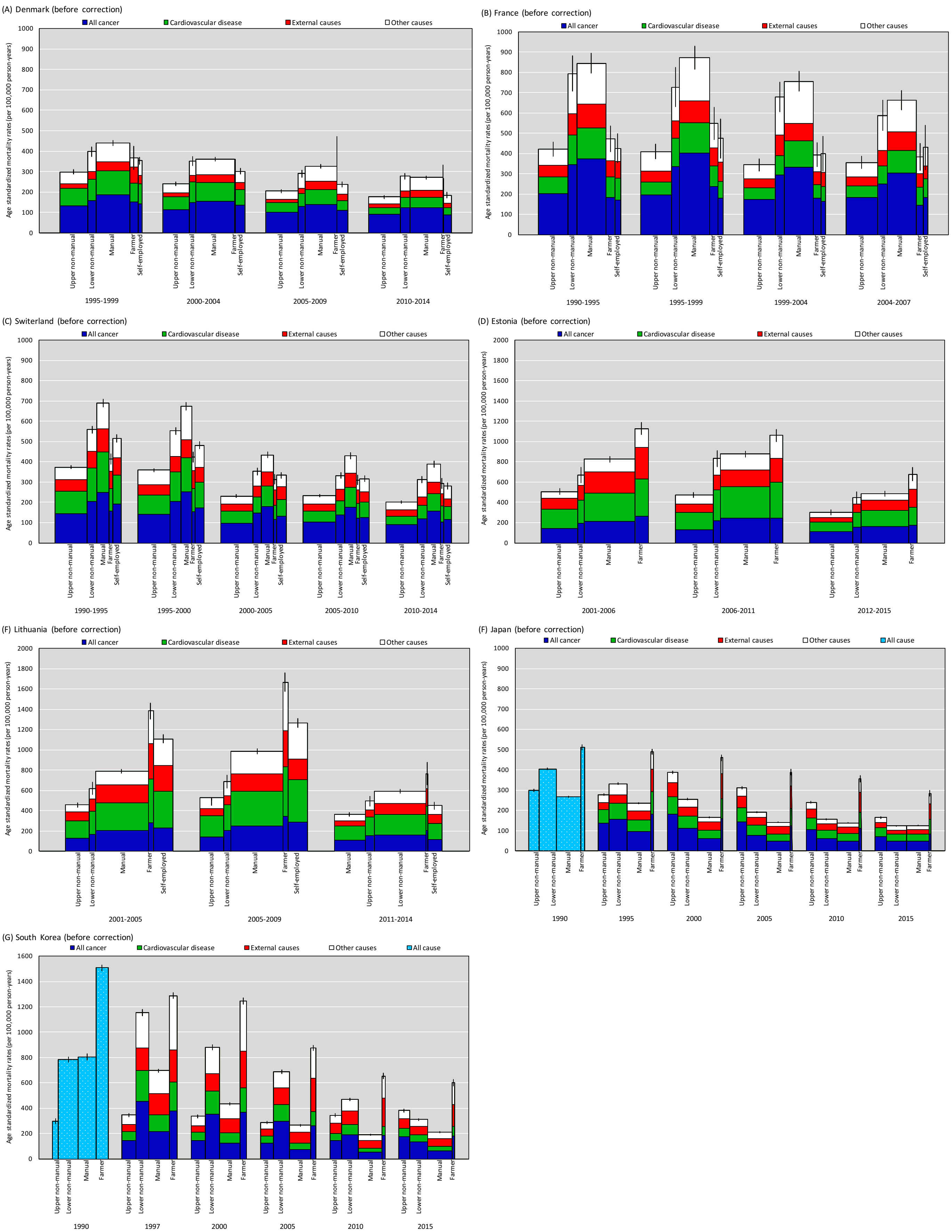

Appendix Figure 4-4. Age-standardized mortality rate and contribution of cause-specific death among men aged 35-64 by countries (before applying correction factor)

Appendix 5 – Sensitivity analysis using an indirect estimation of mortality among the self-employed in Japan and South Korea

Employment status (employed versus self-employed) is not registered on the death certificate in Japan and South Korea, and it was therefore impossible to calculate mortality rates for the self-employed in these two countries, in contrast to most European countries where the self-employed could be distinguished as a separate occupational class. Because this could have biased our results, we conducted a sensitivity analysis, in which we indirectly estimated the mortality rates for the self-employed in Japan and South Korea, and removed the estimated numbers of deaths and person-years of the self-employed in Japan and South Korea from the other occupational classes. We also re-estimated the average inter-group difference (AID absolute and relative version) for five occupational class (upper non-manual, lower non-manual, manual, farmer, and self-employed) using the new estimates for Japan and South Korea. In order to estimate mortality among the self-employed in Japan and South Korea, we proceeded as follows;

1. Percentages of self-employed by occupational class in Japan and South Korea were obtained from the census (in contrast to the death certificate, the census did register employment status; shown in Appendix Table 2-3). These percentages were used to estimate population numbers and person-years for the self-employed in Japan and South Korea, and these were then subtracted from the person-years for the other occupational classes.
2. We estimated the rate ratio of mortality among the self-employed as compared to the whole population in European countries from the available datasets (Appendix Table 5-1). This showed that this rate ratio has an average value of around 0.80. We assumed that the same rate ratio applies in Japan and South Korea.
3. We re-estimated mortality by occupational class using the results of steps 1 and 2 as follows;

$$Modified\ mortality\ rate_z = \frac{(Observed\ mortality\ rate_z - 0.8 * mortality\ rate_{all\ population} * proportion_{z,self-employed})}{1 - proportion_{z,self-employed}} \qquad \text{(A-5.1)}$$

where $proportion_{z,self\text{-}employed}$ is proportion of self-employed among occupational class *z* (data from Appendix Table 2-3). Here, occupational class *z* refers to upper non-manual, lower non-manual, or manual workers (according to the EGP scheme, the occupational class of farmers should include both employed and self-employed farmers).

Appendix Table 5-1. Rate ratios of mortality among self-employed (with mortality in whole population used as a reference)

	1990-1994	1995-1999	2000-2004	2005-2009	2010-2014
Finland	0.84	0.81	0.80	0.84	0.80
Denmark	-	0.56	0.55	0.49	0.44
England/Wales	-	-	-	0.91	0.86
France	0.62	0.70	0.67	0.80	-
Switzerland	0.92	0.97	0.82	0.88	0.89
Italy(Turin)	0.94	1.02	0.89	1.04	-
Estonia	-	-	-	-	-
Lithuania	-	-	0.92	0.93	0.48

Appendix Figure 5-1 and 5-2 show that levels and trends of mortality by occupational class in Japan and South Korea, re-estimated using the indirectly estimated mortality among the self-employed, were not essentially different as compared to those presented in the main text of this paper. We also calculated the mortality rates for the self-employed in Japan and South Korea using different rate ratios for their mortality as

compared to the whole population, i.e., 0.6, 1.0, and 1.2 instead of 0.8 (step 2). Although this somewhat changed the levels of mortality by occupational class and the AIDs (absolute and relative version), it did not materially change the trends and patterns of mortality inequalities by occupational class in Japan and South Korea (results not shown). These results indicate that the pattern of mortality inequalities by occupational class in Japan and South Korea, including the higher mortality rate of upper non-manual group, is unlikely to be biased by this difference in occupational class classification.

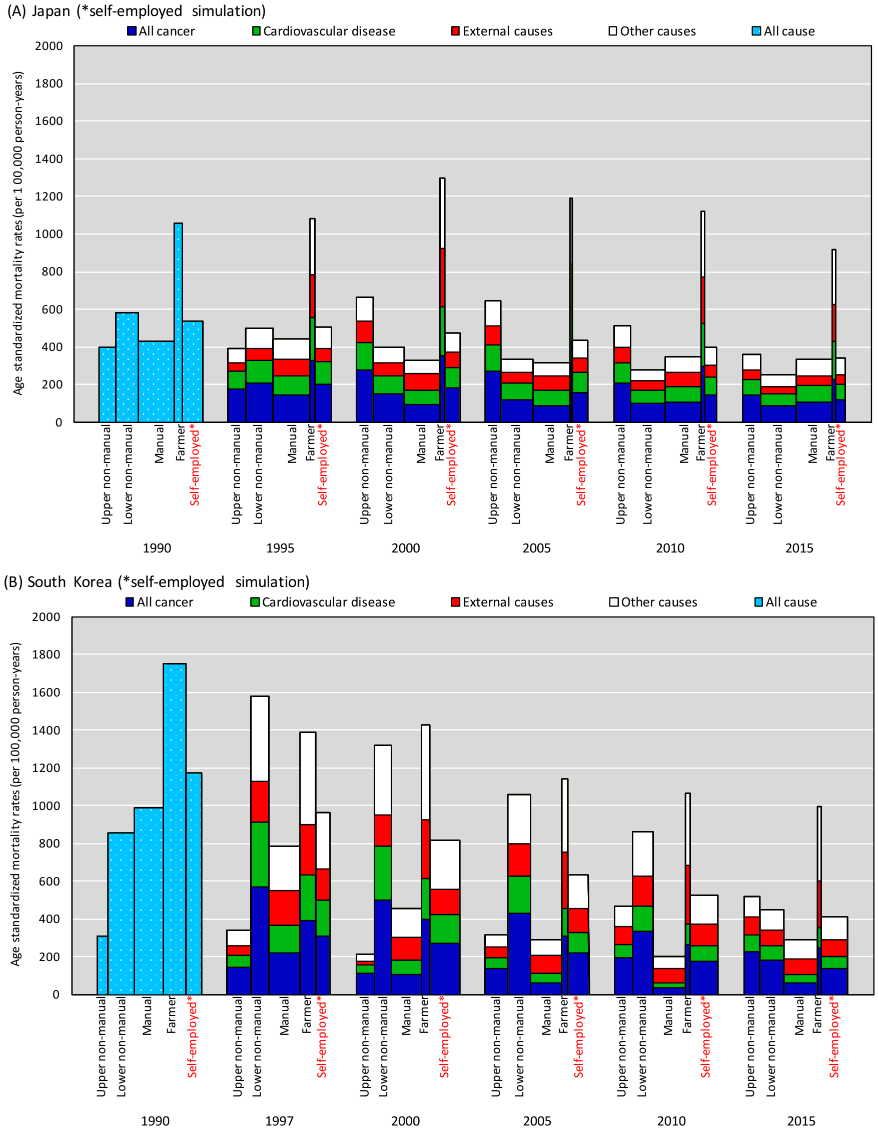

Appendix Figure 5-1. Mortality by occupational class simulating self-employed mortality in Japan and South Korea

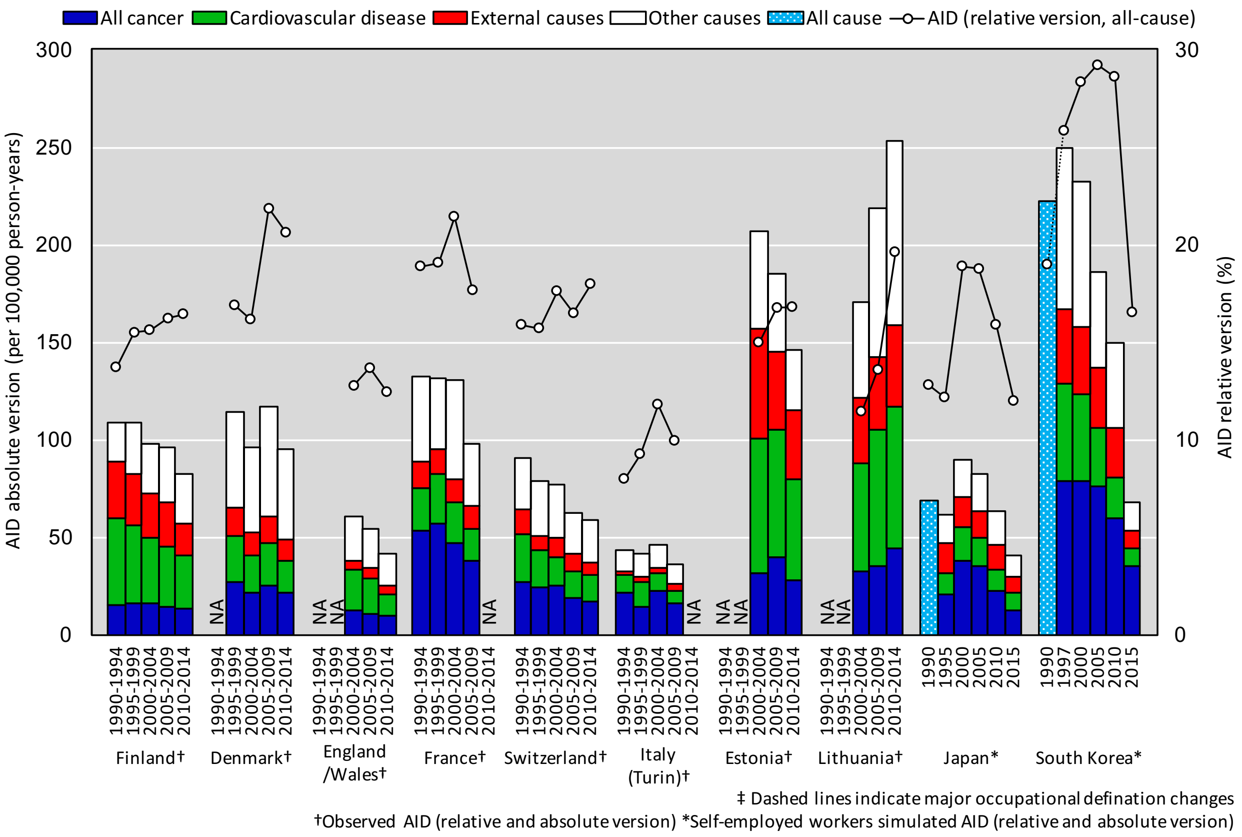

Appendix Figure 5-2. Changes in absolute and relative mortality inequalities (average inter-group difference: AID absolute and relative version) among five occupational class (upper non-manual, lower non-manual, manual, farmer, and self-employed) assuming self-employed mortality in Japan and South Korea, NA; data not available
